# HIGH-DIMENSIONAL NEWEY–POWELL TEST VIA APPROXIMATE MESSAGE PASSING

**DOI:** 10.1017/s0266466626100450

**Published:** 2026-04-21

**Authors:** Jing Zhou, Huı Zou

**Affiliations:** University of Manchester; University of Minnesota

## Abstract

We propose a high-dimensional extension of the heteroscedasticity test proposed in Newey and Powell (1987). Our test is based on expectile regression in the proportional asymptotic regime where n/p→δ∈(0,1]. The asymptotic analysis of the test statistic uses the approximate message passing algorithm, from which we obtain the limiting distribution of the test and establish its asymptotic power. The numerical performance of the test is validated through an extensive simulation study. As real-data applications, we present the analysis based on “international economic growth” data (Belloni et al., 2013), which is found to be homoscedastic, and “supermarket” data (Lan et al., 2016), which is found to be heteroscedastic.

## 1. INTRODUCTION

In many modern economic applications, datasets often contain a large number of covariates p relative to the sample size n. When performing high-dimensional regression on such data, parameter estimation typically assumes homoscedastic errors for simplicity. However, heteroscedasticity is common in practice, and ignoring it can lead to inefficient estimates and invalid inference. Despite its practical importance, testing for heteroscedasticity remains both underexplored and challenging in high-dimensional settings.

Classical heteroscedasticity tests, such as those proposed in Breusch and Pagan (1979), White (1980), and Koenker and Bassett (1982), were developed under the assumption of fixed p, and are no longer valid when p>n, which is the primary focus of this article. To address this gap, we propose a high-dimensional heteroscedasticity test in the proportional regime where n/p→δ∈(0,1]. Our approach extends the classical test of Newey and Powell (1987), which considers the following heteroscedastic linear model:

(1.1) $$Y=X\beta_{0}+\sigma \left(X\gamma_{0}\right)\varepsilon ,$$

where $X=\left(X_{1},\ldots ,X_{p}\right)\in R^{p},\beta_{0},\gamma_{0}\in R^{p}$ are unknown parameter vectors, and Y,ε∈R, with ε independent of X. The function σ(⋅) captures heteroscedasticity, allowing the error variance to vary with the linear combination $X\gamma_{0}$.

To detect nonconstant variance, we use the expectile as a distributional statistic, evaluated at various levels τ∈(0,1), defined as

(1.2) $$u_{\tau }\left(Y\right)-E\left(Y\right)=\frac{2\tau -1}{1-\tau }\int_{\left[u_{\tau }\left(Y\right),\infty \right)}\left(y-u_{\tau }\left(Y\right)\right)dF_{Y}\left(y\right).$$

In model (1.1), the τ th expectile of Y given X is $u_{\tau }(Y|X)=X\beta_{0}+u_{\tau }\left(\sigma \left(X\gamma_{0}\right)\varepsilon \right)$. A key insight is that under heteroscedasticity, the population parameter $\beta_{0}$ is affected by an additional contribution from $\sigma \left(X\gamma_{0}\right)$, scaled by the expectile of the error term ε. In contrast, under homoscedasticity, $\beta_{0}$ remains invariant across different values of τ. This observation becomes even clearer when a local linear approximation of σ(⋅) in (2.3) is considered, and it motivates a testing framework based on distributional statistics (Koenker and Bassett, 1982; Newey and Powell, 1987), where the hypothesis concerns the influence of $\gamma_{0}$.

Compared to previous work (Newey and Powell, 1987), this article addresses two additional challenges commonly encountered in contemporary datasets: (i) the number of parameters p exceeds the sample size n and diverges at a linear rate: n/p→δ∈(0,1] and (ii) the p-vectors $\beta_{0}$ and $\gamma_{0}$ are sparse. This linear rate regime was first discussed in the early approximate message passing (AMP) literature (Donoho, Maleki, and Montanari, 2009, 2010) and then formally stated in Bayati and Montanari (2011a, Thm. 1). The AMP algorithm has attracted significant attention for the theoretical analysis of regularized M-estimators and low-rank matrix estimation. Subsequent work often separately discusses δ∈(0,1] for sparse regression with regularization (e.g., Bradic, 2016; Su, Bogdan, and Candès, 2017; Weinstein, Barber, and Candès, 2017; Weinstein et al., 2023; Zhou, Claeskens, and Bradic, 2020) and δ∈(1,∞) for unstructured regression coefficient vectors (Bradic, 2016; Donoho and Montanari, 2016; Sur, Chen, and Candès, 2019; Sur and Candès, 2019; Candès and Sur, 2020). We refer to Feng et al. (2022) for a thorough literature review. In addition to the AMP framework, El Karoui et al. (2013) and El Karoui (2018) introduced an alternative way of analyzing the estimators in this n,p proportional regime, with conclusions agreeing with the AMP analysis. Specifically, the analysis addresses unregularized and ridge regression, where a sparse structure is not assumed for the parameter vector. In this work, we focus on sparse regression and address the n/p→δ∈(0,1] setting.

In the past decade, fundamental research for high-dimensional linear regression has focused on sparse regularization (Tibshirani, 1996; Candès and Tao, 2007; Zou, 2006; Fan and Lv, 2008; Zhang, 2010). We refer to Bühlmann and van De Geer (2011) and Fan et al. (2020) for a systematic review of this topic. Most existing literature in this field assumes homoscedastic regression errors, that is, σ(⋅) is a constant, as opposed to the fact that heteroscedasticity is commonly observed in real data, see, for example, Wang, Wu, and Li (2012) and Daye, Chen, and Li (2012) on genomic data and Belloni, Chernozhukov, and Wang (2014) on economic data.

There are natural solutions to address heteroscedasticity in model estimation. Cattaneo, Jansson, and Newey (2018) and Jochmans (2022) addressed this by splitting the p covariate vector X into a fixed low-dimensional subvector $X_{s}$ of main interest with unknown parameter $\beta_{s}$ and a nuisance subvector $X_{p-s}$ with nonnegligible size q=p-s. Their high dimensionality refers to a setting where n>p but p is considerably larger than in the classic setting, where p/n→0. The asymptotic normality is further derived for ${\overset{ˆ}{\beta }}_{s}$, and the main focus is on heteroscedastic-robust asymptotic covariance estimators. Addressed by Jochmans (2022, Sect. 3.2, 3.3), the consistency of the heteroscedasticity-robust covariance estimator requires the length of the nuisance parameter to satisfy ${\text{lim}}_{n}\text{sup}\;q/n<1$, and handling the case of $1/2<{\text{lim}}_{n}\text{sup}\;q/n<1$ is already challenging. Another approach considers replacing the least-squares loss function with asymmetric loss functions, such as the quantile loss functions (Koenker and Bassett, 1978; Koenker, 2005) and the expectile loss functions (Newey and Powell, 1987). A thorough review of quantile regression can be found in Koenker (2017). High-dimensional quantile regression, addressing p>n setting, has been studied by several authors (Wu and Liu, 2009; Belloni and Chernozhukov, 2011; Wang et al., 2012; Zheng, Gallagher, and Kulasekera, 2013; Gu et al., 2018; He et al., 2023). Gu and Zou (2016) studied high-dimensional expectile regression and proposed COSALES, which couples two expectile loss functions at expectile level 0.5 and another level of interest. By this construction, COSALES can simultaneously estimate parameter vectors $\beta_{0}$ and $\gamma_{0}$ in (2.3). Additionally, Zhou and Zou (2023) proposed a cross-fitted residual regression for estimating heteroscedasticity in (2.3).

Despite recent efforts in estimating heteroscedasticity through methods, such as quantile regression, expectile regression, or cross-refitted regression, testing for the presence of heteroscedasticity remains largely unexplored in the high-dimensional literature. Since quantile and expectile loss functions are robust to heteroscedastic regression errors, using these two asymmetric loss functions to construct heteroscedastic tests is natural and thus taken in this article. Moreover, as previously mentioned, the Newey–Powell test is shown to be superior to the quantile-based test in low-dimensional settings (Newey and Powell, 1987). Therefore, we focus on the high-dimensional Newey–Powell test in this article. In fact, the contrast between expectile regression and quantile regression is even more significant in high dimensions, owing to the smoother loss function in expectile regression. Gu and Zou (2016) show that fitting a high-dimensional expectile regression model is as simple as fitting Lasso regression. In contrast, serious efforts have been dedicated to handling the nonsmooth loss function in high-dimensional quantile regression, including ADMM (Gu et al., 2018) and smoothing approximation (He et al., 2023).

Given the above considerations, we extend the Newey–Powell test (Newey and Powell, 1987) to sparse high-dimensional settings where n/p→δ∈(0,1], n,p→∞, and $\ell_{1}$-regularization or other sparse regularization is necessary to obtain good estimators. The heteroscedasticity of regression error is tested via $H_{0,j}:\gamma_{0,j}=0$ versus $H_{1,j}:\gamma_{0,j}\neq 0,j=1,\ldots ,p$. By using the AMP theory, we establish the joint asymptotic normality of multiple expectile estimators, which we then use to construct the test statistic. We analytically examine the size and power of the proposed test.

The rest of the article is organized as follows. In Section 2, we first introduce the notation and assumptions for the high-dimensional heteroscedastic linear model in (2.3), following the expectile regression in Section 2.1. The AMP algorithm is introduced in Section 3, and the main technical results are included in Section 3.3. Section 4 presents the proposed heteroscedasticity test. The analytical expression of the power function is presented in Theorem 2. We discuss a practically important decorrelation step for using AMP in applications in Section 5. Section 6 contains simulation studies and real data examples from Belloni et al. (2013) and Lan et al. (2016). Section 7 concludes the article. The assumptions, proofs of lemmas and theorems, and auxiliary lemmas and definitions are provided in Appendixes A–C, respectively. Additional supporting results are included in the Supplementary Material (Zhou and Zou, 2025).

## 2. HETEROSCEDASTIC LINEAR REGRESSION

We restate the well-studied form of the heteroscedastic model from Koenker and Bassett (1982) and Newey and Powell (1987):

(2.3) $$Y=X\beta_{0}+\left(1+X\gamma_{0}\right)\varepsilon ,$$

where $\beta_{0},\gamma_{0}\in R^{p}$ are unknown parameter vectors. In addition, we have n independent and identically distributed (i.i.d.) copies of the pair (Y,X) denoted by $\left(Y_{i},X_{i}\right),i=1,\ldots ,n$. Further, we denote n-vectors $Y={\left(Y_{1},\ldots ,Y_{n}\right)}^{⊤},\varepsilon ={\left(\varepsilon_{1},\ldots ,\varepsilon_{n}\right)}^{⊤}$; the design matrix $X={\left(X_{1},\ldots X_{n}\right)}^{⊤}$. The components of the vector ε satisfy Assumption (A1).

(A1) $\varepsilon_{1},\ldots ,\varepsilon_{n}$ are i.i.d. random variables with mean zero, a finite (2κ-2)th moment for κ≥2.

The constant κ depends on the loss function used to estimate the unknown parameters $\beta_{0},\gamma_{0}$; in our case, the expectile loss function in (2.6). This statement will become clear in Section 3.2 when we discuss the AMP in detail. Further, following Bayati and Montanari (2011a), the empirical measure of a p-vector is defined as putting 1/p weight on each component of the vector. Then, the unknown parameter vector $\gamma_{0}$ complies with Assumption (A2).

(A2) The empirical measure of the components of the p-vector $\gamma_{0}$, when p→∞, converges weakly to the probability measure of a random variable $\Gamma_{0}$ with bounded (2κ-2)th moment for κ≥2.

This assumption precedes a standard Assumption (A3) on the parameter vector $\beta_{0}$ in the AMP literature. In practice, the proposed test is valid for β with finite p components (see Section 4), and the hypothesis in (4.33) tests the components $\gamma_{j}’\text{s}$ for finite p. We also demonstrate applying the proposed test to two real data examples with finite p in Section 6.3.

(A3) The empirical measure of the components of the p-vector $\beta_{0}$, when p→∞, converges weakly to the probability measure of a random variable $B_{0}$ with bounded (2κ-2)th moment for κ≥2.

Notice $X\gamma_{0}=\sqrt{n}X\frac{\gamma_{0}}{\sqrt{n}}$, where the scaled design $\sqrt{n}X$ and parameter vector $\frac{\gamma_{0}}{\sqrt{n}}$ agree with the assumptions in Koenker and Bassett (1982). Further, the sequence $\gamma_{n}=\frac{\gamma_{0}}{\sqrt{n}}$ restricts the calculation to local alternatives and guarantees well approximation of sequences of multiplicative heteroscedastic models.

Following Newey and Powell (1987, Thm. 1(iii)), the τ th expectile of Y given X can be expressed as

(2.4) $$u_{\tau }(Y|X)=X\beta_{0}+\left(1+X\gamma_{0}\right)u_{\tau }(\varepsilon )=X\beta_{\tau }+u_{\tau }(\varepsilon ),$$

where $\beta_{\tau }=\beta_{0}+\gamma_{0}u_{\tau }(\varepsilon )$ is the population parameter vector corresponding to the τ th expectile of Y, approximated by $X\beta_{\tau }$. Here, $u_{\tau }(\varepsilon )$ is the τ th expectile of the error term, as defined in (1.2), and will be denoted simply as $u_{\tau }$ henceforth. The parameter $\beta_{\tau }$ is fixed and depends on the scalar $u_{\tau }$. In this spirit, we rewrite the model as follows:

(2.5) Y=Xβ+ε,

where $\beta =\beta_{0}+\varepsilon \gamma_{0}$ and the error ε satisfies Assumption (A1). In addition, we assume the ratio n/p→(0,1] when n,p→∞. Let $s=\|\beta {\|}_{0}$, where $\|\cdot {\|}_{0}$ denotes the $\ell_{0}$-norm of a vector. Obviously, $s\leq {\left\|\beta_{0}\right\|}_{0}+{\left\|\gamma_{0}\right\|}_{0}$. The way of parameterization in (2.5) has two reasons: (1) we want to express the model to fit it in the AMP framework for asymptotic analysis, where an independent error term stands alone and (2) the error term ε determines the location change of β from $\beta_{0}$. Identifying $\gamma_{0}$ relies on making use of different spots of the error distribution and requires a pair of regularized estimators. This is technically challenging due not only to the nontrivial asymptotic behavior of regularized estimators when p>n, but also to the correlation structure among pairs of estimators whose population parameters do not have the same center.

### 2.1. Expectile Regression under Heteroscedasticity

The expectile regression (Newey and Powell, 1987) estimates the τ th conditional expectile of Y by minimizing an asymmetric quadratic loss function defined as

(2.6) $$\rho_{\tau }\left(x;u_{\tau }\right)=\left|\tau -I\left\{x\leq u_{\tau }\right\}\right|{\left(x-u_{\tau }\right)}^{2},$$

where τ∈(0,1) is the expectile level and $u_{\tau }$ denotes the τ th expectile of error ε. which is the solution to the nonlinear equation (Newey and Powell, 1987, Eq. (2.7)). A special case is τ=1/2, where the expectile loss function reduces to the least-squares loss. Under our model, the conditional expectile of Y at expectile level τ follows $X\left(\beta_{0}+\gamma_{0}u_{\tau }\right)+u_{\tau }$ (Newey and Powell, 1987) and the coefficient vector $\beta_{\tau }=\beta_{0}+\gamma_{0}u_{\tau }$, where $u_{\tau }$ is the expectile of error ε at τ in (1.2). Since ε has mean zero (Assumption (A1)), expectile regression at τ=1/2 does not identify the combination of $\gamma_{0}$ and $\beta_{0}$. Instead, $\beta_{1/2}$ only estimates $\beta_{0}$.

The proposed heteroscedasticity test is constructed based on the estimator of the parameter vector β via minimizing the empirical expectile loss function. Specifically, we consider the $\ell_{1}$-regularized expectile estimator defined as follows:

(2.7) $${\hat{\beta }}_{\tau }(\lambda )=\text{arg}\;\underset{\beta \in R^{p}}{\text{min}}\sum_{i=1}^{n}\rho_{\tau }\left(Y_{i}-X_{i}\beta ;u_{\tau }\right)+\lambda \|\beta {\|}_{1},$$

where $\|\cdot {\|}_{1}$ denotes the $\ell_{1}$-norm and λ denotes the tuning parameter. Due to technical restrictions of the AMP algorithm (see Section 3), we do not jointly optimize the intercept term $u_{\tau }$ and the slope vector β. Instead, we treat the term $u_{\tau }$ as a parameter of the expectile loss function. Recentering Y by the intercept $u_{\tau }$ is important to guarantee the following AMP analysis when considering robust loss functions. In our simplest homoscedastic error case, plugging in $u_{\tau }$ reduces the conditional τ-mean in (2.4) to only $X\beta_{\tau }$. This intercept term is only ignorable when we address the conditional mean of Y given X by Assumption (A1), the zero mean of ε. In homoscedastic settings, the error expectile $u_{\tau }$ can be estimated by solving

(2.8) $${\overset{ˆ}{u}}_{\tau }-\frac{1}{n}\sum_{i=1}^{n}r_{i}=[(2\tau -1)/(1-\tau )]\frac{1}{n}\sum_{i=1}^{n}\left(r_{i}-{\overset{ˆ}{u}}_{\tau }\right)I\left\{r_{i}\geq {\overset{ˆ}{u}}_{\tau }\right\},$$

where $r_{i}=Y_{i}-{\hat{\beta }}_{\tau }X_{i}’\text{s}$ are obtained using an initial estimator $\hat{\beta }$, such as the regularized least-squares estimators, the regularized Huber estimator, etc. However, estimating $u_{\tau }$ becomes challenging under heteroscedasticity since solving (2.8) estimates the τ’s expectile of $\sigma \left(X\gamma_{0}\right)\varepsilon$ instead of ε. We observe that $u_{\tau }$ is reflected in the intercept term of $\gamma_{0}u_{\tau }$ in the composition $\beta_{\tau }=\beta_{0}+\gamma_{0}u_{\tau }$. Since the coupled sparse asymmetric least squares (COSALES) (Gu and Zou, 2016) estimates $\beta_{0}$ and $\gamma_{0}u_{\tau }$ simultaneously for any τ∈(0,1),τ≠1/2, we use the intercept estimator of $\gamma_{0}u_{\tau }$ from the COSALES as a surrogate for the true $u_{\tau }$ in (1.2). Further, to evaluate the estimation error of $u_{\tau }$ on the test, we compared the numerical results using the true and estimated $u_{\tau }$. We observe that using the estimated $u_{\tau }$ from the COSALES does not affect the test results. We also investigated the performance of the test when plugging in (2.8) under the null in Table S.1 in the Supplementary Material (Zhou and Zou, 2025). The numerical performance appears similar.

Importantly, when the regression errors are i.i.d., that is, σ(⋅)=1, the slope β does not vary across different expectile levels in the classical setting with fixed p and n→∞. We propose to test the violation of homoscedasticity under diverging n,p based on the ground of common slope under homoscedasticity at different expectile levels $\tau_{k},k=1,\ldots ,K$.

## 3. APPROXIMATE MESSAGE PASSING FOR EXPECTILE REGRESSION

The AMP analysis starts by regularizing the loss functions ρ by using the proximal mapping operator defined as

(3.9) $$\text{Prox}\;(z;b)=\text{arg}\;\underset{x\in R}{\text{min}}\left\{b\rho (x)+\frac{1}{2}(x-z{)}^{2}\right\},$$

where the parameter b>0 is a positive scalar that controls the step size moving toward the minimum of the loss function ρ. In our case, the proximal operator for expectile loss (2.6) can be obtained following the derivation in Section B.1 and can be expressed as follows:

(3.10) $${\text{Prox}\;}_{\tau }(z;b)=\left\{\begin{array}{ll} \left(z+2b(1-\tau )u_{\tau }\right)(2b(1-\tau )+1{)}^{-1}, & z\leq u_{\tau }\\ \left(z+2b\tau u_{\tau }\right)(2b\tau +1), & z>u_{\tau } \end{array}\right.\;=\frac{z-2bu_{\tau }\left(\tau -I\left\{z\leq u_{\tau }\right\}\right)}{1-2b\left(\tau -I\left\{z\leq u_{\tau }\right\}\right)}.$$

With the analytical expressions of the proximal operator for the expectile loss function (3.10), we further work out the expression of the effective score function (Donoho and Montanari, 2016) defined as

(3.11) $$\tilde{G}(z;b)={\left.b\partial \rho \left(x\right)\right|}_{x=\text{Prox}\left(z;b\right)},\;\text{with}\;b>0,$$

where ∂ρ is the subgradient of ρ defined as ∂ρ(x)={y:ρ(u)≥ρ(x)+y(u-x),forallu}. This leads to the subgradient of the expectile loss function in (3.12) as

(3.12) $$\partial \rho_{\tau }\left(z\right)=2\left|I\left\{z\leq u_{\tau }\right\}-\tau \right|\left(z-u_{\tau }\right).$$

One could interpret the effective score function by associating it with the score function. By plugging (3.10) in (3.12) and using the definition of the effective score function in (3.11), we obtain the effective score function as follows:

(3.13) $${\tilde{G}}_{\tau }(z;b)=\left\{\begin{array}{ll} \frac{2b(1-\tau )}{2b(1-\tau )+1}\left(z-u_{\tau }\right), & z\leq u_{\tau }\\ \frac{2b\tau }{2b\tau +1}\left(z-u_{\tau }\right), & z>u_{\tau }. \end{array}\right.$$

To analyze regularized estimators under a sparse structure, specifically, when s/p→ω, where s denotes the number of nonzero components of β, Bradic (2016) and Zhou et al. (2020) incorporated sparsity by introducing the rescaled effective score function

(3.14) $$G\left(z;b\right)=\delta \omega^{-1}\tilde{G}\left(z;b\right).$$

### 3.1. The Approximate Message Passing Algorithm

We consider the AMP algorithm to approximate the estimators in (2.7) in the sense that the averaged difference between the estimators obtained from the AMP algorithm and the estimators by minimizing the loss functions converges to zero almost surely, see Zhou et al. (2020, p. 2562, Eq. (23) and below) for a summary of discussions in the relevant references (Bayati and Montanari, 2011b; Bradic, 2016; Donoho and Montanari, 2016; Sur et al., 2019; Huang, 2020).

The AMP algorithm is an iterative algorithm indexed by t consisting of three steps that belong to the general recursion in Bayati and Montanari (2011a). We will describe the general recursion when stating the theory in Section 3.3. Let the matrix X satisfy Assumption (A4).

(A4) A standard Gaussian design: $X_{i},i=1,\ldots ,n$, are i.i.d. copies of X, where $X^{⊤}\in R^{p}$ with $X_{j}\sim N(0,1/n),j=1,\ldots ,p$, i.i.d. components.

Since $\beta_{\tau }$ is a sparse vector with most components being zero, the algorithm starts with an initialization ${\hat{\beta }}_{\left(0\right)}=0,t=0$ and proceed at t≥1 following steps:

**Step 1. Adjust residuals** The adjusted residuals $z_{\left(t\right)}$ are obtained by
(3.15) $$z_{\left(t\right)}=Y-X{\hat{\beta }}_{\left(t\right)}+n^{-1}G\left(z_{\left(t-1\right)};b_{\left(t-1\right)}\right)\sum_{j=1}^{p}I\left\{\eta \left({\hat{\beta }}_{(t-1),j}+X_{j}G\left(z_{\left(t-1\right)};b_{\left(t-1\right)}\right);\theta_{\left(t-1\right)}\right)\neq 0\right\},$$
  where G is defined in (3.14) and $\eta \left(\cdot ;\theta_{\left(t-1\right)}\right)$ is the soft-thresholding operator with parameter $\theta_{\left(t-1\right)}$ defined as η(β;θ)=sgn(β)max(|β|-θ,0).
**Step 2. Update the parameter for effective score function** The scalar parameter $b_{\left(t\right)}$ is chosen such that the average of the effective score function has slope 1, that is, $\frac{1}{n}\sum_{i=1}^{n}\partial_{1}G\left(z_{i,(t)};b_{\left(t\right)}\right)=1$, where $\partial_{1}G(z;b)$ denotes the derivative of G w.r.t. the first argument z in case of differentiable function G. Hence, for the expectile regression with calculations in Section B.2, the parameter $b_{\left(t\right)}$ is updated by solving
(3.16) $$\frac{\omega }{\delta }=\frac{2b(1-\tau )}{2b(1-\tau )+1}\frac{\#\left\{i:z_{(t),i}\leq u_{\tau }\right\}}{n}+\frac{2b\tau }{2b\tau +1}\frac{\#\left\{i:z_{(t),i}>u_{\tau }\right\}}{n},$$
  where ω is estimated by the fraction of the nonnull components in an initial estimator $\hat{\beta }$, and $u_{\tau }$ is replaced by plugging in an estimator from the COSALES algorithm (see discussions under (2.8)). The update $b_{\left(t\right)}$ at step t is taken to be the first value on a fine grid that solves (3.16).
**Step 3. Update the estimators for** β Update the coefficient estimator for β as follows:
(3.17) $${\hat{\beta }}_{\left(t+1\right)}=\eta \left({\tilde{\beta }}_{\left(t\right)};\theta_{\left(t\right)}\right),\;\text{where}\;{\tilde{\beta }}_{\left(t\right)}={\hat{\beta }}_{\left(t\right)}+X^{⊤}G\left(z_{\left(t\right)};b_{\left(t\right)}\right).$$
  The estimator $\hat{\beta }$ is an approximation for regularized estimators in (2.7), where the soft-thresholding operator η(⋅;θ) is the proximal operator for the regularizer $\|\beta {\|}_{1}$. The tuning parameter θ is chosen to minimize the mean squared error (MSE) of $\hat{\beta }$. As suggested in Donoho and Johnstone (1994, 1998), Donoho et al. (2009), and Bayati and Montanari (2011b), we take $\theta_{\left(t\right)}=\alpha {\overset{‾}{\zeta }}_{\text{emp}(t)}$ for scalar α and
(3.18) $${\overset{‾}{\zeta }}_{\text{emp},(t)}^{2}=\frac{1}{n}\sum_{i=1}^{n}G{\left(z_{i,(t)};b_{\left(t\right)}\right)}^{2}.$$
  In practice, one chooses a grid of values for α, then obtains the corresponding estimator $\hat{\beta }$ and the estimated MSE for each value of α on the grid. The optimal α is chosen to be the one with the minimum estimated MSE. The details can be found in Zhou et al. (2020, Algorithm 1). We also implement practical stopping rules: the maximum number of iterations is set to T=100, and convergence is declared when $\text{MSE}\left({\hat{\beta }}_{\left(t+1\right)},{\hat{\beta }}_{\left(t\right)}\right)=\frac{1}{p}\sum_{j=1}^{p}{\left({\hat{\beta }}_{j,(t+1)}-{\hat{\beta }}_{j,(t)}\right)}^{2}<\text{tol}$. In the simulations, the tolerance level tol is set to 10^−8^ for high sparsity settings and 10^−8^ for medium sparsity settings.
  Details of the population version of the parameter ${\overset{‾}{\zeta }}_{\text{emp},(t)}^{2}$ can be found in Section 3.2. The estimator $\tilde{\beta }$ is of major interest in this project since it is connected to the debiased estimator (Javanmard and Montanari, 2014; van de Geer et al., 2014) and follows a Gaussian distribution asymptotically (Bayati and Montanari, 2011b; Bayati, Erdogdu, and Montanari, 2013). We will discuss this in Section 3.3.

### 3.2. State Evolution

When p,n→∞, the averaged-over-components performance of estimators ${\tilde{\beta }}_{\left(t\right)}$ and ${\hat{\beta }}_{\left(t\right)}$ from the AMP algorithm can be described using two important parameters ${\overset{‾}{\zeta }}_{\left(t\right)}^{2},{\overset{‾}{\sigma }}_{\left(t\right)}^{2}$. Assume that the following limit exists ${\overset{‾}{\sigma }}_{\left(0\right)}^{2}={\text{lim}}_{p\to \infty }(p\delta {)}^{-1}{\left\|q_{\left(0\right)}\right\|}^{2}<\infty$. With such initialization, the state evolution recursion for t∈{0}⋃N is defined as

(3.19) $${\overset{‾}{\zeta }}_{\left(t\right)}^{2}=E\left[G{\left(\varepsilon +{\overset{‾}{\sigma }}_{\left(t\right)}Z;b_{\left(t\right)}\right)}^{2}\right],$$

(3.20) $${\overset{‾}{\sigma }}_{\left(t\right)}^{2}=\delta^{-1}E\left[{\left(\eta \left(B+{\overset{‾}{\zeta }}_{\left(t-1\right)}Z;\theta_{\left(t-1\right)}\right)-B\right)}^{2}\right],$$

where B denotes the limiting form of the components of β in (2.5) and Z~N(0,1) is a standard Gaussian random variable that is independent of B and ε. The parameter ${\overset{‾}{\zeta }}_{\left(t\right)}$ is the limiting version of (3.18) when n,p→∞ (Zhou et al., 2020, Sect. B.2.3).

The readers might wonder if (3.19) and (3.20) still hold under heteroscedasticity since the relevant literature has not discussed this issue and often assumes homoscedasticity. It is worth mentioning that the state evolution recursion in (3.19) and (3.20) is valid as long as the empirical distribution of ε and the components of β converge weakly (Bayati and Montanari, 2011a, Thm. 2). We will discuss this in detail in Section 3.3.

Using the two parameters, we can analyze the limiting performance of the estimator $\hat{\beta }$ from the AMP algorithm. A typical example is analyzing the asymptotic MSE (AMSE), defined as $\text{AMSE}\left({\hat{\beta }}_{\left(t+1\right)},\beta \right)={\text{lim}}_{p\to \infty }\frac{1}{p}\sum_{j=1}^{p}{\left({\hat{\beta }}_{(t+1),j}-\beta_{j}\right)}^{2}$. It is easy to show that AMSE complies with

$$\text{AMSE}\left({\hat{\beta }}_{\left(t+1\right)},\beta \right)=\underset{p\to \infty }{\text{lim}}\frac{1}{p}\sum_{j=1}^{p}{\left(\eta \left({\tilde{\beta }}_{(t),j};\theta_{\left(t\right)}\right)-\beta_{j}\right)}^{2}\overset{\text{a.s.}}{=}E\left[{\left\{\eta \left(B+{\overset{‾}{\zeta }}_{\left(t\right)}Z;\theta_{\left(t\right)}\right)-B\right\}}^{2}\right],$$

which corresponds to Bradic (2016, Eq. (3.4)) with ${\tilde{\beta }}_{(t),j}$ in (3.17). An important observation from (3.20) is that, at each iteration step t, the estimator ${\tilde{\beta }}_{(t),j}$ is obtained by adding a Gaussian noise $N\left(0,{\overset{‾}{\zeta }}_{\left(t\right)}^{2}\right)$ to the jth component of the true parameter vector $\beta_{j}$. This suggests that ${\tilde{\beta }}_{\left(t\right)}\sim N\left(\beta ,{\overset{‾}{\zeta }}_{\left(t\right)}^{2}I_{p\times p}\right)$ for finite marginals. Similar results are also obtained for the Lasso estimator in Bayati and Montanari (2011b), which is a special case of the $\ell_{1}$-regularized expectile regression when τ=1/2. We will come back to the asymptotic normality of ${\tilde{\beta }}_{\left(t\right)}$ in Section 3.3 after introducing the main technical lemma for multiple estimators ${\tilde{\beta }}_{k,(t)},k=1,1\ldots ,K$.

### 3.3. Asymptotic Analysis for Multiple Estimators

The heteroscedasticity test for expectile regression is based on the difference of the estimators at different expectile levels $\tau_{k},k=1,\ldots ,K$. The test statistic is based on asymptotic analysis for multiple estimators ${\hat{\beta }}_{k}’\text{s}$ among which the correlation is nontrivial when estimated using the same dataset (Zhou et al., 2020, Lemma 1). Since estimating the correlation requires information on the unknown parameter vector β, we further extend the discussion on the correlations to the adjusted residuals $z_{k,(t)}’\text{s}$ in the main technical result—Lemma 1 and construct an adjusted-residual-based estimator for the covariance between ${\tilde{\beta }}_{k}’\text{s}$. Further, we want to address that the technical results, including the general recursion and Lemma 1 in this section, hold for similar loss functions, such as the quantile loss function.

We describe the general recursions in Bayati and Montanari (2011a) for the completeness of the article and a better connection of Lemma 1 and the AMP algorithm in Section 3.1. Under the heteroscedastic model (2.5), the coefficient vector $\beta_{k}=\beta_{0}+u_{k}\gamma_{0}$, where $u_{k}$ is a constant featuring the strength of heteroscedasticity which depends on ε. For expectile regression, $u_{k}$ is the error expectile. By Assumptions (A2) and (A3), the empirical distribution of the parameter vector $\beta_{k}$ converges weakly to $B_{k}=B_{0}+u_{k}\Gamma_{0}$. In cases where $u_{k}=0$ or homoscedasticity such that $\Gamma_{0}=0$ degenerates, $B_{k}=B_{0}$.

In addition, let a noise vector $\varepsilon \in R^{n}$ and the coefficient vector $\beta_{k}\in R^{p}$ satisfy Assumptions (A1) and (A3). By fixing the initial condition $q_{k,(0)}$ and recall the convention $m_{k,(-1)}=0$, the general recursion for t≥0 is defined as

(3.21) $$h_{k,(t+1)}=X^{⊤}m_{k,(t)}-\xi_{1,k,(t)}q_{k,(t)},\;m_{k,(t)}=g_{1,k,(t)}\left(d_{k,(t)},\varepsilon \right),\;d_{k,(t)}=Xq_{k,(t)}-\xi_{2,k,(t)}m_{k,(t-1)},\;q_{k,(t)}=g_{2,k,(t)}\left(h_{k,(t)},\beta_{k}\right),$$

where

(3.22) $$\xi_{1,k,(t)}=n^{-1}\sum_{i=1}^{n}\partial_{1}g_{1,k,(t)}\left(d_{k,(t),i},\varepsilon_{i}\right),$$

(3.23) $$\xi_{2,(t)}=(\delta p{)}^{-1}\sum_{j=1}^{p}\partial_{1}g_{2,k,\left(t\right)}\left(h_{k,\left(t\right),j},\beta_{k,j}\right).$$

As a special case, the AMP algorithm takes

$$h_{k,(t+1)}=\beta_{k}-X^{⊤}G_{k}\left(z_{k,\left(t\right)};b_{k,\left(t\right)}\right)-{\hat{\beta }}_{k,\left(t\right)},$$

$$q_{k,(t)}={\hat{\beta }}_{k,(t)}-\beta_{k},\;z_{k,\left(t\right)}=\varepsilon -d_{k,\left(t\right)},$$

which indicates

(3.24) $$d_{k,(t)}=\varepsilon -z_{k,\left(t\right)},\;m_{k,\left(t\right)}=-G_{k}\left(z_{k,\left(t\right)};b_{k,\left(t\right)}\right),$$

with the functions $g_{1,k,(t)}\left(x_{1},x_{2}\right)=-G_{k}\left(x_{2}-x_{1};b_{k,(t)}\right)$ and $g_{2,k,(t)}\left(x_{1},\beta \right)=\eta (\beta -\left.x_{1};\theta_{k,(t)}\right)-\beta$. Notice ${\hat{\beta }}_{k,(t)}$ the regularized estimator in (3.17), and

(3.25) $$h_{k,(t+1)}=\beta_{k}-{\tilde{\beta }}_{k,\left(t\right)}.$$

This detail is mathematically trivial but allows us to apply Lemma 1 directly to obtain the asymptotic normality of any pairs $\left({\tilde{\beta }}_{k_{1},(t)},{\tilde{\beta }}_{k_{2},(t)}\right)$, as well as later obtain the power function for the proposed hypothesis test in Theorem 2.

Before presenting the main technical lemma, we assume a moment condition (Assumption (A5)), which also complements Assumptions (A1)–(A3). Recall in Assumptions (A1)–(A3), we state that the empirical measures of the components of the vectors $\beta_{0},\gamma_{0}$, and ε converge weakly to the probability measures of random variables $B_{0},\Gamma_{0}$, and ε, respectively. Let ${\hat{f}}_{\beta_{0}(p)},{\hat{f}}_{\gamma_{0}(p)}$, and ${\hat{f}}_{\varepsilon (p)}$ denote the empirical measures of $\beta_{0},\gamma_{0}$, and ε, that is, putting equal weights on the components of the vectors. Specifically, for the sequence of vectors ε(p), we index the sequence by p to unify the notation following Bayati and Montanari (2011a). This sequence index is legit since our asymptotic theory is derived under n/p→δ∈(0,1]. Then, Assumption (A5) can be stated as follows:

(A5) For some κ>1, let the empirical measures ${\hat{f}}_{\beta_{0}(p)},{\hat{f}}_{\gamma_{0}(p)}$, and ${\hat{f}}_{\varepsilon (p)}$ converge weakly to the probability measures $f_{B_{0}},f_{\Gamma_{0}}$, and $f_{\varepsilon }$. In addition, assume the following (2κ-2) th moment conditions hold:
  - ${\text{lim}}_{p\to \infty }E_{{\hat{f}}_{\beta_{0}(p)}}\left(B_{0}^{2\kappa -2}\right)=E_{f_{B_{0}}}\left(B_{0}^{2\kappa -2}\right)<\infty$;
  - ${\text{lim}}_{p\to \infty }E_{{\hat{f}}_{\gamma_{0}(p)}}\left(\Gamma_{0}^{2\kappa -2}\right)=E_{f_{\Gamma_{0}}}\left(\Gamma_{0}^{2\kappa -2}\right)<\infty$;
  - ${\text{lim}}_{p\to \infty }E_{{\hat{f}}_{\varepsilon (p)}}\left(\varepsilon^{2\kappa -2}\right)=E_{f_{\varepsilon }}\left(\varepsilon^{2\kappa -2}\right)<\infty$;
  - ${\text{lim}}_{p\to \infty }E_{{\hat{f}}_{q_{k}(p)}}\left(B_{k}^{2\kappa -2}\right)<\infty$.

The constant κ depends on the pseudo-Lipschitz order of function $g_{1,k,(t)}$, see Definition 1 for the definition of pseudo-Lipschitz functions and Lemma 1 for associating the order κ with the function $g_{1}={\tilde{\psi }}_{c^{\prime }},{\tilde{\psi }}_{c^{\prime \prime }}$. As described above, in the special case where the general recursion (3.21) is the AMP algorithm, function $g_{1}$ takes a more specific form, that is, the negative effective score function $-G_{k}(\cdot ;\cdot )$.

Lemma 1. *Let the sequences of design matrices* {X(p)}, *location coefficient vectors $\left\{\beta_{0}(p)\right\}$*, *heteroscedasticity parameter vectors* $\left\{\gamma_{0}(p)\right\}$, *error vectors* {ε(p)}, *and initial condition vectors* $\left\{q_{k}(p)\right\}$ *be weakly convergent sequences for* K *recursions satisfying Assumptions (A1)–(A5). Let* $\left\{{\overset{‾}{\sigma }}_{k,(t)}^{2},{\overset{‾}{\zeta }}_{k,(t)}^{2}\right\}$ *be defined uniquely by the recursions in* (3.19) *and* (3.20). *These are the state evolution parameters for the* k*th estimation with initialization* ${\overset{‾}{\sigma }}_{k,(0)}^{2}={\text{lim}}_{n\to \infty }(p\delta {)}^{-1}{\left\|q_{k,(0)}\right\|}^{2}$. *Further, we assume a constant* $u_{k}$ *for each of the* K *recursions specifying the signal strength of the heteroscedasticity parameter, such that the parameter vector* (2.5) *has the composition* $\beta_{k}=\beta_{0}+u_{k}\gamma_{0}$. *Assume Lemma 1 in* Bayati and Montanari (2011a) *holds individually for each of the* K *recursions. Additionally, for all pseudo-Lipschitz functions* ${\tilde{\psi }}_{c}:R^{t+2}\to R$ *of order* $\kappa_{c}$ *for some* $1\leq \kappa_{c}=\kappa /2$ *with* κ *as in (A5) and the iteration index* t≥0,t∈Z,

(3.26) $$\underset{p\to \infty }{\text{lim}}\frac{1}{p}\overset{p}{\underset{j=1}{\sum }}{\tilde{\psi }}_{\text{c}}\left(h_{k_{1},(1),j},\ldots ,h_{k_{1},(t+1),j},\beta_{k_{1},j}\right){\tilde{\psi }}_{\text{c}}\left(h_{k_{2},(1),j},\ldots ,h_{k_{2},(t+1),j},\beta_{k_{2},j}\right)\overset{\text{a.s.}}{=}\;E\left[{\tilde{\psi }}_{\text{c}}\left({\overset{‾}{\zeta }}_{k_{1},(0)}Z_{k_{1},(0)},\ldots ,{\overset{‾}{\zeta }}_{k_{1},(t)}Z_{k_{1},(t)},B_{k_{1}}\right){\tilde{\psi }}_{\text{c}}\left({\overset{‾}{\zeta }}_{k_{2},(0)}Z_{k_{2},(0)},\ldots ,{\overset{‾}{\zeta }}_{k_{2},(t)}Z_{k_{2},(t)},B_{k_{2}}\right)\right].$$

*And, for all pseudo-Lipschitz functions ${\tilde{\psi }}_{{\text{c}}^{\prime }},{\tilde{\psi }}_{{\text{c}}^{\prime \prime }}:R^{t+2}\to R$ of order* $\kappa_{{\text{c}}^{\prime }},\kappa_{{\text{c}}^{\prime \prime }}$ *for some* $1\leq \kappa_{{\text{c}}^{\prime }},\kappa_{{\text{c}}^{\prime \prime }}\leq \kappa /2$, *where* $\kappa_{c}^{\prime }+\kappa_{c}^{\prime \prime }=\kappa$ *with* κ *as in (A5)*,

(3.27) $$\underset{n\to \infty }{\text{lim}}\frac{1}{n}\sum_{i=1}^{n}{\tilde{\psi }}_{c^{\prime }}\left(d_{k_{1},\left(0\right),i},\ldots ,d_{k_{1},\left(t\right),i},\varepsilon_{i}\right){\tilde{\psi }}_{c^{\prime \prime }}\left(d_{k_{2},\left(0\right),i},\ldots ,d_{k_{2},\left(t\right),i},\varepsilon_{i}\right)\overset{\text{a.s.}}{=}\;E[{\tilde{\psi }}_{c^{\prime }}\left({\bar{\sigma }}_{k_{1},\left(0\right)}{\hat{Z}}_{k_{1},\left(0\right)},\ldots ,{\bar{\sigma }}_{k_{1},\left(t\right)}{\hat{Z}}_{k_{1},\left(t\right)},\varepsilon \right){\tilde{\psi }}_{c^{\prime \prime }}\left({\bar{\sigma }}_{k_{2},\left(0\right)}{\hat{Z}}_{k_{2},\left(0\right)},\ldots ,{\bar{\sigma }}_{k_{2},\left(t\right)}{\hat{Z}}_{k_{2},\left(t\right)},\varepsilon \right)],$$

*where* $\left(Z_{k,(0)},\ldots ,Z_{k,(t)}\right),\left({\overset{ˆ}{Z}}_{k,(0)},\ldots ,{\overset{ˆ}{Z}}_{k,(t)}\right)$ *are multivariate Gaussian with*, $k=k_{1},k_{2}$, *is a* (t+1)-*dimensional zero-mean multivariate standard normal vector independent of $B_{k},\varepsilon$*; *at iteration* $t,\left(Z_{k_{1},(t)},Z_{k_{2},(t)}\right)$ *and* $\left({\overset{ˆ}{Z}}_{k_{1},(t)},{\overset{ˆ}{Z}}_{k_{2},(t)}\right)$ *are standard normal 2-vectors with covariance not necessarily equal to zero*.

*Further, for $0<t_{1},t_{2}<t$*, *with* $\overset{P}{\to }$ *denoting convergence in probability, it holds that*

(3.28) $$\frac{1}{n}\sum_{i=1}^{n}m_{k_{1},\left(t_{1}\right),i}m_{k_{2},\left(t_{2}\right),i}\overset{P}{\to }\underset{p\to \infty }{\text{lim}}\frac{1}{p}\sum_{j=1}^{p}h_{k_{1},\left(t_{1}+1\right),j}h_{k_{2},\left(t_{2}+1\right),j}.$$

Lemma 1 generalizes Bayati and Montanari (2011a, Lemma 1(b)) by considering the correlation structure between multiple recursions. Further, Lemma 1 extends the analysis to account for error heteroscedasticity, which has not been addressed in the relevant literature. When $k_{1}=k_{2}$ and the heteroscedastic parameter vector $\gamma_{0}=0$ is a null vector, Lemma 1 reduces to Bayati and Montanari (2011a, Lemma 1(b)). Further, the covariance matrices of the standard normal 2-vectors $\left(Z_{k_{1},(t)},Z_{k_{2},(t)}\right)$ and $\left({\overset{ˆ}{Z}}_{k_{1},(t),}{\overset{ˆ}{Z}}_{k_{2},(t)}\right)$ will be involved in constructing the test statistic in Section 4. The proof of Lemma 1 is provided in the Supplementary Material (Zhou and Zou, 2025).

**Remark 1.** We thank a reviewer for drawing our attention to Theorem 1 and Lemma 2 in Javanmard and Montanari (2013). Their result agrees with our conclusion in (3.26), which shows the asymptotic approximation of the covariance of the structure of multiple estimators. Our additional contributions lie in establishing (3.27) and (3.28), which are of independent interest to statistics and econometrics, as they are important for establishing Theorem 1, (3.30), and demonstrate that the correlation between estimators is linked to the correlation of residuals.

To guarantee an estimable test statistic, we propose an adjusted-residual-based estimator in Theorem 1, which is directly obtainable from the AMP algorithm. The construction of the covariance matrix estimators is included in Section B.4 by directly applying (3.28). It is worth noting that (3.28) is a contribution to the existing AMP literature. The significance of (3.28) is that it shows the correlation between estimators (noticing $h_{k,(t+1)}=\beta -{\tilde{\beta }}_{k,(t)}$ in (3.25)) from two sequences is asymptotically equivalent to the product of two effective score functions. This avoids the problem of lacking information about the true parameter vector β. Additionally, Theorem 1 validates that $Z_{k_{1}}$ and $Z_{k_{2}}$ are correlated by (3.29).

Theorem 1. *Under the assumptions in Lemma 1 and* κ=2 *for any two recursions $k_{1},k_{2}=1,\ldots ,K$*, *with probability 1, it holds that*

(3.29) $$\underset{p\to \infty }{\text{lim}}\frac{1}{p}\sum_{j=1}^{p}\left({\tilde{\beta }}_{k_{1},(t),j}-\beta_{k_{1},j}\right)\left({\tilde{\beta }}_{k_{2},(t),j}-\beta_{k_{2},j}\right)={\overset{‾}{\zeta }}_{k_{1},(t)}{\overset{‾}{\zeta }}_{k_{2},(t)}\text{Cov}\left(Z_{k_{1}},Z_{k_{2}}\right),$$

(3.30) $$\underset{n\to \infty }{\text{lim}}\frac{1}{n}\sum_{i=1}^{n}G_{k_{1}}\left(z_{k_{1},(t),i};b_{k_{1},(t)}\right)G_{k_{2}}\left(z_{k_{2},(t),i};b_{k_{2},(t)}\right)=E\left[G_{k_{1}}\left(\varepsilon +{\overset{‾}{\sigma }}_{k_{1}}{\overset{ˆ}{Z}}_{k_{1}};b_{k_{1},(t)}\right)G_{k_{2}}\left(\varepsilon +{\overset{‾}{\sigma }}_{k_{2}}{\overset{ˆ}{Z}}_{k_{2}};b_{k_{2},(t)}\right)\right].$$

*The estimator constructed as follows:*

(3.31) $${\hat{\zeta }}_{k_{1},k_{2},(t)}=\frac{1}{n}\overset{n}{\underset{i=1}{\sum }}G_{k_{1}}\left(z_{k_{1},(t),i};b_{k_{1},(t)}\right)G_{k_{2}}\left(z_{k_{2},(t),i};b_{k_{2},(t)}\right).$$

*is a consistent estimator of the covariance ${\overset{‾}{\zeta }}_{k_{1},(t)}{\overset{‾}{\zeta }}_{k_{2},(t)}\text{Cov}\left(Z_{k_{1},(t)},Z_{k_{2},(t)}\right)$*.

Theorem 1 first validates our claim in Lemma 1 that $Z_{k_{1},(t),}Z_{k_{2},(t)}$ are correlated. The correlation between $Z_{k_{1},(t)},Z_{k_{2},(t)}$ describes the averaged correlation between the components of ${\tilde{\beta }}_{k_{1},(t)}$ and ${\tilde{\beta }}_{k_{2},(t)}$. A similar conclusion holds for ${\overset{ˆ}{Z}}_{k_{1},(t)},{\overset{ˆ}{Z}}_{k_{2},(t)}$. Further, the covariance between $Z_{k_{1},(t),}Z_{k_{2},(t)}$ can be approximated by using the adjusted residuals without any information about β.

The last piece of information before moving to the test statistic is the joint asymptotic distribution of the pair $\left({\tilde{\beta }}_{k_{1}},{\tilde{\beta }}_{k_{2}}\right)$.

Lemma 2 (Joint normality). *Under the assumptions in Lemma 1 for any two recursions* $k_{1},k_{2}=1,\ldots ,K$ *and for any pseudo-Lipschitz function* ψ:R→R *of order* $\kappa_{c}$ *in* (3.26), *the following convergence for estimators from the AMP algorithm holds with probability 1*:

(3.32) $$\underset{p\to \infty }{\text{lim}}\frac{1}{p}\overset{p}{\underset{j=1}{\sum }}\psi \left({\tilde{\beta }}_{k_{1},(t),j}\right)\psi \left({\tilde{\beta }}_{k_{2},(t),j}\right)=E\left[\psi \left(B_{k_{1}}+{\overset{‾}{\zeta }}_{k_{1},(t)}Z_{k_{1}}\right)\psi \left(B_{k_{2}}+{\overset{‾}{\zeta }}_{k_{2},(t)}Z_{k_{2}}\right)\right],$$

*where* $\left(Z_{k_{1}},Z_{k_{2}}\right)$ *is a standard normal random 2-vector*.

The proof of Lemma 2 is a direct application of Lemma 1 (see Section B.3). When writing in the matrix form, the conditional joint asymptotic normality $\tilde{\xi }-\xi \sim N\left(\left(u_{k_{1}},u_{k_{2}}\right)⊗\gamma_{0},\Sigma_{\left(t\right)}⊗I_{p\times p}\right)$ holds for $k_{1},k_{2}=1,\ldots ,K$, where $\tilde{\xi }=\left({\tilde{\beta }}_{k_{1},(t)},{\tilde{\beta }}_{k_{2},(t)}\right),\xi =(1,1)⊗\beta_{0}$, and $I_{p\times p}$ is a p×p identity matrix. The covariance matrix $\Sigma_{\left(t\right)}$ has $(k_{1},k_{2})\text{th}$ component $\Sigma_{k_{1},k_{2},(t)}={\overset{‾}{\zeta }}_{k_{1},k_{2},(t)}$, which can be estimated by (3.31).

Under homoscedasticity where $\gamma_{0}=0,{\tilde{\beta }}_{k_{1}}$ and ${\tilde{\beta }}_{k_{2}}$ have a common center $\beta_{0}$. In addition, variance components ${\overset{‾}{\zeta }}_{k,(t)}^{2},k=1,\ldots ,K$, are inflated under heteroscedasticity. This phenomenon can be observed by the inflation of the standard deviation ${\overset{‾}{\sigma }}_{k,(t)}^{2}$ in (3.19), which is caused by the extra heteroscedastic parameter in (3.20).

## 4. HETEROSCEDASTICITY TEST

Although the asymptotic normality of $\tilde{\xi }$ holds for all iterations t=0,1,… of the AMP algorithm, we use the estimators $\tilde{\xi }$ at t→∞ to construct test statistics. When the iteration t→∞, the $l_{2}$-norm of the difference of the AMP output and the estimator in (2.7) converges to zero almost surely (see Bradic 2016, Thm. 2). In practice, we consider a stopping rule and define the algorithm's convergence in Section 3.1 under (3.18). At the stop, we obtain an estimator approximating the estimator defined in (2.7). Hence, the iteration index t is dropped in the following sections. We consider testing the components of the vector $\gamma_{0}$

(4.33) $$H_{0,j}:\gamma_{0,j}=0\;\text{versus}\;H_{1,j}:\gamma_{0,j}\neq 0,j=1,\ldots ,p.$$

Lemma 2 states that the K expectile estimators follow a multivariate Gaussian distribution asymptotically with a common center $\beta_{0}$ under homoscedasticity. And the rows of $\tilde{\xi }$ are i.i.d. following a joint Gaussian distribution with covariance matrix Σ.

For $\gamma_{0,j}$, the test statistic is defined as

(4.34) $$T_{j}=\frac{\Delta {\tilde{\xi }}_{j}^{⊤}}{\sqrt{\Delta \Sigma \Delta^{⊤}}},j=1,\ldots ,p.$$

Following Newey and Powell (1987, Sect. 4.1), we consider the contrast between two expectile levels for the testing problem, that is, $\tilde{\xi }=\left({\tilde{\beta }}_{\tau_{1}},{\tilde{\beta }}_{\tau_{2}}\right),\Delta =(1,-1)$, and the components of the covariance matrix Σ are replaced by their estimators in (3.31). Then, by Lemma 2, the test statistic $T_{j}$ follows a standard normal distribution N(0,1). For the two-sided alternative in (4.33), the p-value can be calculated by

(4.35) $$P_{j}=2\left\{1-\Phi \left(\left|\frac{\Delta {\tilde{\xi }}_{j}^{⊤}}{\sqrt{\Delta \Sigma \Delta^{⊤}}}\right|\right)\right\},$$

with rejection region $R=\left(-\infty ,\Phi^{-1}(\alpha /2)\right)\cup \left(\Phi^{-1}(1-\alpha /2),\infty \right)$, where Φ denotes the cumulative distribution function of a standard normal distribution N(0,1). The null hypothesis is rejected if $P_{j}\leq \alpha$ at nominal level α. To analyze the power function on the population scale when n,p→∞, we consider for any jth component $\vartheta \left(\gamma_{0,j}\right)=I\left(T_{j}\in R,\Gamma_{0}=\gamma_{0,j}\right)$. Then, the limiting expression of the function ϑ is obtained in Theorem 2 with proof in Appendix B.5.

Theorem 2. *The function denoted by* $\vartheta \left(\gamma_{0,j}\right),j=1,\ldots ,p$ *complies with*

(4.36) $$\underset{p\to \infty }{\text{lim}}\frac{1}{p}\sum_{j=1}^{p}\vartheta \left(\gamma_{0,j}\right)\overset{\text{a.s.}}{=}\;E_{\Gamma_{0}}\left[1-\Phi \left(\Phi^{-1}(1-\alpha /2)-\Gamma_{0}\frac{\Delta {\left(u_{\tau_{1}},u_{\tau_{2}}\right)}^{⊤}}{\sqrt{\Delta \Sigma \Delta^{⊤}}}\right)+\Phi \left(\Phi^{-1}(\alpha /2)-\Gamma_{0}\frac{\Delta {\left(u_{\tau_{1}},u_{\tau_{2}}\right)}^{⊤}}{\sqrt{\Delta \Sigma \Delta^{⊤}}}\right)\right].$$

Theorem 2 can be used to confirm the size and power of the proposed test. Assume the nominal significance level of the test is α. The size of the test $P\left(T_{j}\in \right.R|\Gamma_{0}=0$) is approximated by

$$\frac{{\text{lim}}_{p\to \infty }\frac{1}{p}\sum_{j=1}^{p}I\left(T_{j}\in R,\Gamma_{0}=0\right)}{P\left(\Gamma_{0}=0\right)}\;\overset{\text{a.s.}}{=}\frac{E_{\Gamma_{0}}\left[1-\Phi \left(\Phi^{-1}(1-\alpha /2)-0\right)+\Phi \left(\Phi^{-1}(\alpha /2)-0\right)|\Gamma_{0}=0\right]P\left(\Gamma_{0}=0\right)}{P\left(\Gamma_{0}=0\right)}\;=\alpha ,$$

where the conditional expectation is obtained by (4.36) at $\Gamma_{0}=0$. This confirms that the size of a nominal α test is α asymptotically.

Further, the empirical size of the test is defined in (6.37) and used to evaluate the performance of the proposed test. As presented in Tables 1 and 2, the empirical size, averaged over the components of $\gamma_{0}$, is approximately equal to the nominal level α. Similarly, the power of the test is obtained by considering the nonnull components $\gamma_{0,j}’\text{s}$. However, from the right-hand side of (4.36), we notice that the power of the test depends on the averaged signal strength of $\Gamma_{0}$, the expectiles $\left(u_{\tau_{1}},u_{\tau_{2}}\right)$, and the covariance matrix Σ. The empirical power of the test defined in (6.38) is also used to evaluate the numerical performance of the test in Section 6.

## 5. DECORRELATION IN AMP

It is well known that the standard Gaussian design assumption, which is stated in Assumption (A4), is important for the AMP framework. Researchers have made several attempts to relax this assumption. One direction is to relax the Gaussian distribution assumption. For example, Bayati, Lelarge, and Montanari (2015) and Chen and Lam (2021) showed that the AMP framework could be extended to other random matrices; Rangan, Schniter, and Fletcher (2019) and Fan (2022) discussed the extension of AMP algorithms to right rotationally invariant design. Li and Sur (2026) showed the statistical applications of these algorithms. However, the independence assumption is still essential in these works. A more relevant direction for this work is to relax the independence assumption in order to handle general design matrices with arbitrary covariance matrices. Consider a general design matrix in the sparse high-dimensional heteroscedastic linear model: $\tilde{Y}=\tilde{X}\beta +\tilde{\varepsilon }$, where $\tilde{Y}\in R^{n},\tilde{X}\in R^{n\times p},\tilde{\varepsilon }\in R^{n}$, and the rows of $\tilde{X}$ are i.i.d. from a multivariate Gaussian distribution $N_{p}(0,\tilde{\Sigma })$. Several papers have discussed incorporating a general Gaussian design with strictly positive-definite covariance matrices. For example, Donoho and Montanari (2016, Def. 4.5, Cor. 4.6), Candès and Sur (2020, Sect. 3.1.1), Zhao et al. (2022, Sect. 2), and Huang (2022) have proposed a method to decorrelate the design matrix using the nonsingular covariance matrix $\tilde{𝚺}$ by applying the transformation $\left(\tilde{X}{\tilde{𝚺}}^{-1/2}\right)/\sqrt{n}$. This decorrelation allows for satisfying Assumption (A4) and expands the applicability of AMP-based methods and theories. However, this decorrelation approach has limitations when dealing with sparse parameter vectors β because the null components of β become nonnull under the linear transformation $\sqrt{n}{\tilde{𝚺}}^{1/2}\beta$ and the corresponding back transformation. Additionally, in practical applications, the covariance matrix $\tilde{𝚺}$ is often unknown, and estimating it becomes challenging when p>n unless certain structure assumptions are made. These challenges restrict the use of this approach in real applications.

In applications, we consider a different method for decorrelation in the AMP. The validity of this type of decorrelation has been tested numerically in Zhou and Claeskens (2023, Table 3). We utilize a “generalized puffer transformation” on the data, resulting in transformed variables denoted as $Y=F\tilde{Y}$ and $X=F\tilde{X}$, where the transformed data satisfy the model $Y=X\beta +F\tilde{\varepsilon }$. It is important to note that in our setting, p>n. To perform the transformation, we utilize matrices $U\in R^{n\times n},D\in R^{n\times n}$, and $V^{⊤}\in R^{n\times p}$ obtained from the singular value decomposition $\tilde{X}=UDV^{⊤}$. The “generalized puffer transformation” was originally proposed in Jia and Rohe (2015), where the authors aimed to decorrelate the design matrix to bypass the so-called “irrepresentable condition” for the Lasso regression. The “generalized puffer transformation” uses $F=\sqrt{p/n}U\overset{ˆ}{D}U^{⊤}$, where $\overset{ˆ}{D}$ is a diagonal matrix with diagonal elements ${\overset{ˆ}{D}}_{ii}=I\left(D_{ii}\leq 1/\sqrt{n}\right)\sqrt{n}+I\left(D_{ii}>1/\sqrt{n}\right)/D_{ii},i=1,\ldots ,n$. Subsequently, we apply the AMP algorithm and conduct the hypothesis test using the transformed data (Y,X).

Unlike the decorrelation step in Donoho and Montanari (2016), Candès and Sur (2020), and Zhao et al. (2022), our approach does not involve a transformation of β. Thus, it can preserve sparse structures. When p>n, we cannot guarantee that X precisely satisfies Assumption (A4), but the correlation is largely reduced, as observed in the numerical study. Moreover, on the transformed data, the error term becomes $F\tilde{\varepsilon }$. Under the null hypothesis that the model (2.3) is homoscedastic, we have verified that $F\tilde{\varepsilon }$ is independent of the transformed design matrix $X=\sqrt{p/n}UV^{⊤}$. This observation suggests that the “generalized puffer transformation” does not alter the null hypothesis. This explains why the proposed test retains its validity size, as demonstrated in our simulation study. For further details, please refer to Section 6.2.

## 6. NUMERICAL PERFORMANCE

### 6.1. Simulation

We consider simulation settings where p=500 and n=250. The samples $X_{i}$ are generated from a standard Gaussian design, as specified in Assumption (A4). The nonnull components of the true parameter vector $\beta_{0}$ are generated using a standard normal N(0,1) distribution and then multiplied by 5. Each setting is replicated R=400 times. The parameter vector $\beta_{0}$ and design matrix X are fixed across R replications using the same seed number, while the regression errors ε vary in each replication. We evaluate the test under six different error distributions: $N(0,1),t_{3}$, Lognormal (0, 1), 0.9N(-0.2,0.25)+0.1N(1.8,0.01), 0.9N(0.2,0.25)+0.1N(-1.8,0.01), and 0.95N(0,0.25)+0.05N(0,4). The first three error distributions are randomly generated, centered, and scaled to have a standard deviation of 0.5. We aim to assess the test's performance using symmetric errors (represented by N(0,1)), heavy-tailed errors (represented by $t_{3}$), and fattailed errors (represented by Lognormal (0, 1)). Additionally, we consider three mixture normal distributed errors. The first two, 0.9N(-2,0.25)+0.1N(18,0.01) and 0.9N(2,0.25)+0.1N(-18,0.01), model clustered outliers on the right and left sides of the main error distribution, respectively. The last distribution—0.95N(0,0.25)+0.05N(0,4)—adds additional variability to N(0,0.25). For heteroscedasticity, we assume five components of $\gamma_{0}$ are nonnulls $(3,1,-5,-5,-3{)}^{⊤}$, and the rest components are all zeros. Similar to $\beta_{0}$, the components of $\gamma_{0}$ are fixed throughout R replications using the same seed number.

The individual test is evaluated by the averaged size (or false positive) proportion and power (or true positive proportion). Both proportions are obtained by averaging over simulation replications and the components of $\gamma_{0}$, which agrees with the construction of the power function in Theorem 2. At significance level α, the size can be estimated via

(6.37) $$\text{FP}(\alpha )=(p-s{)}^{-1}\sum_{j\in S^{\text{c}}}\sum_{r=1}^{R}I\left\{P_{r,j}\leq \alpha \right\}/R\;\text{(empirical size)},$$

and the power can be estimated by

(6.38) $$\text{TP}(\alpha )=s^{-1}\sum_{j\in S}\sum_{r=1}^{R}I\left\{P_{r,j}\leq \alpha \right\}/R\;\text{(empirical power)}.$$

For homoscedastic cases, the components of $\gamma_{0}$ are all zeros and ${\left\|\gamma_{0}\right\|}_{0}=0$; hence only FP values are calculated. For heteroscedasticity cases, five components of $\gamma_{0}$ are nonnull with magnitude $(3,1,-5,-5,-3{)}^{⊤}$. The signal strength of heteroscedasticity $u_{\tau }\gamma_{0}$ depends on the error expectile $u_{\tau }$. Since the errors are scaled to a standard deviation of 0.5, the heteroscedasticity signal strength is largely weakened by $u_{\tau }$.

We consider two sparsity levels for fixed ratio δ=0.5,p=500, and n=250.

1. (High-sparsity) $\beta_{0}$ has s=5 nonnull components.
2. (Medium-sparsity) $\beta_{0}$ has s=40 nonnull components.

The above two settings intend to investigate the impact of sparsity. Further, we consider homoscedastic and heteroscedastic regression errors for each sparsity level, where the errors are generated from six different distributions. Simulation results are presented in Tables 1 and 2. The test statistic in (4.34) is constructed using pairs of expectile estimators $\xi =\left({\tilde{\beta }}_{\tau_{1}},{\tilde{\beta }}_{\tau_{2}}\right)$, where seven pairs—$\tau_{2}=0.8,\;\tau_{1}=0.1,0.2,0.6$, and $\tau_{2}=0.9,\tau_{1}=0.1,0.2,0.6,0.8$—are tested. Since the test results for four pairs are similar, we only report two pairs—$\left(\tau_{1},\tau_{2}\right)=(0.2,0.8)$ and (0.6, 0.8). Table 1 uses expectile levels at $\left(\tau_{1},\tau_{2})=(0.2,0.8\right)$, and Table 2 uses expectile levels at $\left(\tau_{1},\tau_{2}\right)=(0.6,0.8)$.

In addition, under homoscedasticity of the regression error, the test statistic in (4.34) can be seen as samples from a standard normal N(0,1) distribution. To confirm the theory, we also inspected the normality of the test statistic constructed using different pairs of expectile estimators. Example QQ-plots are included in Figure S.1 in the Supplementary Material (Zhou and Zou, 2025); the examples are randomly chosen among simulation replications to avoid “cherry-picking.”

We make several observations from Tables 1 and 2. When comparing Tables 1 and 2, we observe that using the expectile level pair $\left(\tau_{1},\tau_{2}\right)=(0.2,0.8)$ leads to higher test power, which agrees with Theorem 2 that shows the power of the test depends on the difference $\left(u_{\tau_{2}}-u_{\tau_{1}}\right)$. Placing two expectile levels far enough would preserve the signal strength of $\gamma_{0}$ to a certain extent. However, we do not suggest using two very extreme expectiles, since estimating them is more challenging than estimating those closer to the center.

When comparing the results for different error distributions, we note that the average FP proportions are close to the nominal α, which complies with Theorem 2. The average FP proportions are slightly greater than the nominal level for the mixture normal distribution 0.9N(-0.2,0.25)+0.1N(1.8,0.01). At the same time, this error distribution tends to have the highest empirical power among all six error distributions. In medium-sparsity settings, where the number of nonnull components of $\beta_{0}$ is 40, estimating $\beta_{\tau }$ becomes more challenging, and it is not surprising that the power of the test in this setting dropped compared to Setting 2, where the number of nonnull components of $\beta_{0}$ is 5. The estimation variances diminish the signal strength when s=40. This setting is very challenging for all tests, not just for ours.

### 6.2. Check the Decorrelation Proposal

We use simulations to check the validity of the decorrelation step proposed in Section 5. We assume an autoregressive (AR(1)) covariance matrix for $\tilde{X}\sim N(0,\tilde{𝚺})$, where the components ${\tilde{𝚺}}_{ij}=\rho^{\left|i-j\right|}$ for i,j=1,…,p. The error distributions considered are N(0,1) and $t_{3}$. To assess the impact of correlation on test performance, we consider ρ=0.3 and 0.7.

We see in Tables 3 and 4 that the test is still valid numerically using the transformed data by the “generalized puffer transformation.” We have checked that on the transformed data, the error is independent of the covariates, which suggests that the null hypothesis remains unchanged after the transformation. Simulation results demonstrate that the size of the test is approximately equal to the nominal α. The power of the test decreases as the “generalized puffer transformation” reduces the signal-to-noise ratio. Furthermore, the power of the test decreases when the correlation parameter ρ increases from 0.3 to 0.7.

### 6.3. Real Data Examples

#### 6.3.1. International Economic Growth Data.

We apply the proposed test on the “international economic growth” data used in Belloni et al. (2013), where the authors mainly focused on variable selection and estimation. We now want to test whether heteroscedasticity is present in this data. The dataset is available on the website https://stuff.mit.edu/~vchern/NBER/ and consists of n=90 samples. Further, we exclude the intercept term in the unprocessed dataset. The original dataset consists of 61 covariates measuring aspects, such as education, trading openness, and science policies. We further expand the covariate set by including the polynomial terms up to order 4 of all 61 covariates. The expanded dataset consists of 244 covariates with a ratio δ approximately 0.37. The primary interest is the response variable—the national growth rate of GDP per capita. We apply the decorrelation procedure outlined in Section 5 before constructing the test. Similar to Section 6.1, the test statistic is calculated using two $\ell_{1}$-regularized expectile estimators. We consider expectile estimators at level $\tau_{2}=0.8$ and at $\tau_{1}=0.2$. According to Belloni et al. (2013), the dataset is homoscedastic since no unmeasured underlying variables exist. Our test results show that none of the covariates cause heteroscedasticity across the different pairs. To assess the stability of the method when using pairs at different expectile levels, we also test $\tau_{1}=0.4,0.5,0.6$. The test results agree with $\tau_{1}=0.2$ for this dataset.

#### 6.3.2. Supermarket Data.

We also apply the proposed heteroscedasticity test to the “supermarket data” used in Lan et al. (2016). The authors extended the t-test in their study and focused on testing $\beta_{0,j}\neq 0$ in a high-dimensional setting (p≫n). The dataset consists of n=464 daily records, where the response variable is the number of customers, and the covariates represent the sales volume of 6,398 different products. Although the dataset is potentially heteroscedastic, the test proposed in Lan et al. (2016) is robust to heteroscedastic errors.

Similarly, we utilize the fused Kolmogorov filter (Mai and Zou, 2015) to reduce the number of covariates to p=928, achieving δ=n/p=0.5. The ratio δ=n/p=0.5. Note that this reduced dimension is well beyond the theoretical limit O(n/log(n)), which ensures the sure screening property of the fused Kolmogorov filter. Thus, we have high confidence that the screening step only removes noisy features and retains all important features. After applying the decorrelation procedure, we employ a pair of $\ell_{1}$-regularized expectile estimators at $\left(\tau_{1},\tau_{2}\right)=(0.2,0.8)$ to construct the test. The test results indicate that 61 covariates potentially contribute to heteroscedasticity. We also tested $\tau_{1}=0.4,0.5,0.6$ and $\tau_{2}=0.8$. When using the pair $\left(\tau_{1},\tau_{2}\right)=(0.6,0.8)$, potentially 43 covariates contribute to heteroscedasticity. In addition, there are 34 common covariates detected by both $\tau_{1}=0.2$ and $\tau_{1}=0.6$. The other two pairs using $\tau_{1}=0.4,0.6$ have more stable performance, overlapping 43 and 47 covariates with the pair using $\tau_{1}=0.2$.

## 7. CONCLUDING REMARKS

In this article, we propose a high-dimensional Newey–Powell heteroscedasticity test based on contrasting expectile regression at two different expectile levels. The testing procedure is based on a bias-corrected estimator directly obtained from the AMP algorithm in Section 3, which is tracked by the state evolution recursion asymptotically when n,p→∞. We clarify that we want to offer an alternative debiasing framework in addition to the existing one, which requires adding a bias-correction term to the regularized estimators. Such a well-explored debiasing framework requires estimating sparse sample precision matrices and a thorough theoretical investigation for the bias-corrected estimator, which requires a considerable amount of work. The proposed test has stable performance when $\gamma_{0}$ is highly sparse, meaning that only a few covariates $X_{j}$ contribute to the heteroscedasticity. The numerical performance suggests that the error distributions also have an impact on the size and power of the test, which is worth further investigation. Although the AMP theory is developed for multiple levels, we did not explore contrasting multiple expectiles for testing heteroscedasticity in this work. This could be interesting for future research.

Upon examining Theorem 2, we observe that the strength of the signal in $\gamma_{0}$ and the covariance of the two expectile estimators also impact the power of the test. An open question remains regarding the optimal choice of expectile levels.

While the current literature on the AMP framework assumes an independent Gaussian design matrix, it is important to acknowledge that this assumption can be relaxed. However, a significant challenge in AMP remains unresolved: the lack of known theories to relax the assumption of independent covariates, accounting for both numerical and theoretical aspects. It is crucial to emphasize that this challenge is not unique to our work but is a broader issue in the AMP literature. To address this limitation in practical applications, we propose a “generalized puffer transformation” to enhance the applicability of the proposed test to real-world data. Through a simulation study, we demonstrate that the resulting test maintains its validity by preserving its nominal size. However, a further theoretical investigation is necessary to understand and address this challenge, and it remains an open question for future research.

## Supplementary Material

Supplementary

Zhou, J., & Zou, H. (2025). Supplement to “High-dimensional Newey–Powell test via approximate message passing,” Econometric Theory Supplementary Material. To view, please visit: https://doi.org/10.1017/S0266466626100450.

## Figures and Tables

**Table 1.** Test (α=0.05) results for different distributions of ε under homoscedastic and heteroscedastic variance

$\alpha =0.05,\tau_{1}=0.2,\tau_{2}=0.8$									
ε	Homoscedastic			Heteroscedastic					
	N(0,1)	$t_{3}$	Lognormal(0, 1)	N(0,1)	$t_{3}$	Lognormal(0, 1)	N(0,1)	$t_{3}$	Lognormal(0, 1)
	FP(size)			FP(size)			TP(power)		
High-sparsity	0.05	0.05	0.05	0.05	0.05	0.05	0.50	0.32	0.34
Medium-sparsity	0.06	0.06	0.05	0.05	0.06	0.06	0.09	0.09	0.09
ε	Homoscedastic			Heteroscedastic					
	mixNormal 1	mixNormal 2	mixNormal 3	mixNormal 1	mixNormal 2	mixNormal 3	mixNormal 1	mixNormal 2	mixNormal 3
	FP(size)			FP(size)			TP(power)		
High-sparsity	0.06	0.05	0.05	0.06	0.05	0.05	0.60	0.32	0.34
Medium-sparsity	0.06	0.05	0.06	0.06	0.05	0.07	0.19	0.08	0.10

*Note:* The pair of expectile levels is $\left(\tau_{1},\tau_{2}\right)=(0.2,0.8)$. The top half presents results for ε following $N(0,1),t_{3}$, Lognormal(0,1); the bottom half for 0.9N(-0.2,0.25)+0.1N(1.8,0.01) (mixNormal 1), 0.9N(0.2,0.25)+0.1N(-1.8,0.01) (mixNormal 2), 0.95N(0,0.25)+0.05N(0,4) (mixNormal 3). Each simulation setting is replicated R=400 times. The average FP proportions are calculated for homoscedastic variance, where the heteroscedastic parameter vector $\gamma_{0}=0$. For heteroscedastic variance, where $\gamma_{0}$ has five nonnull components (3,1, −5, −5, −3), the average FP and TP proportions are calculated.

**Table 2.** Test (α=0.05) results for different distributions of ε under homoscedastic and heteroscedastic variance

$\alpha =0.05,\tau_{1}=0.6,\tau_{2}=0.8$									
ε	Homoscedastic			Heteroscedastic					
	N(0,1)	$t_{3}$	Lognormal(0, 1)	N(0,1)	$t_{3}$	Lognormal(0, 1)	N(0,1)	$t_{3}$	Lognormal(0, 1)
	FP(size)			FP(size)			TP(power)		
High-sparsity	0.05	0.05	0.05	0.05	0.05	0.05	0.24	0.17	0.17
Medium-sparsity	0.06	0.06	0.05	0.06	0.06	0.06	0.05	0.06	0.06
ε	Homoscedastic			Heteroscedastic					
	mixNormal 1	mixNormal 2	mixNormal 3	mixNormal 1	mixNormal 2	mixnormal 3	mixNormal 1	mixNormal 2	mixnormal 3
	FP(size)			FP(size)			TP(power)		
High-sparsity	0.05	0.04	0.05	0.05	0.04	0.04	0.28	0.13	0.16
Medium-sparsity	0.06	0.04	0.04	0.06	0.04	0.06	0.10	0.05	0.09

*Note:* The pair of expectile levels is $\left(\tau_{1},\tau_{2}\right)=(0.6,0.8)$. The top half presents results for ε following $N(0,1),t_{3}$, Lognormal(0,1); the bottom half for 0.9N(-0.2,0.25)+0.1N(1.8,0.01) (mixNormal 1), 0.9N(0.2,0.25)+0.1N(-1.8,0.01) (mixNormal 2), 0.95N(0,0.25)+0.05N(0,4) (mixNormal 3). Each simulation setting is replicated R=400 times. The average FP proportions are calculated for homoscedastic variance, where the heteroscedastic parameter vector $\gamma_{0}=0$. For heteroscedastic variance, where $\gamma_{0}$ has five nonnull components (3,1,-5,-5,-3), the average FP and TP proportions are calculated.

**Table 3.** Test (α=0.05) results are presented for the decorrelation procedure under both homoscedastic and heteroscedastic variance

$\alpha =0.05,\tau_{1}=0.2,\tau_{2}=0.8$												
ε	ρ=0.3						ρ=0.5					
	Homoscedastic		Heteroscedastic				Homoscedastic		Heteroscedastic			
	N(0,1)	$t_{3}$	N(0,1)	$t_{3}$	N(0,1)	$t_{3}$	N(0,1)	$t_{3}$	N(0,1)	$t_{3}$	N(0,1)	$t_{3}$
	FP(size)		FP(size)		TP(power)		FP(size)		FP(size)		TP(power)	
High-sparsity	0.05	0.05	0.05	0.05	0.40	0.26	0.05	0.05	0.05	0.05	0.33	0.23
Medium-sparsity	0.06	0.05	0.06	0.06	0.13	0.13	0.07	0.07	0.06	0.06	0.12	0.11

*Note:* The pair of expectile levels is $\left(\tau_{1},\tau_{2}\right)=(0.2,0.8)$. The covariance matrix for $\tilde{X}\sim N(0,\tilde{𝚺})$ has components ${\tilde{𝚺}}_{ij}=\rho^{\left|i-j\right|}$ for i,j=1,…,p. The error distributions considered are N(0,1) and $t_{3}$. Each simulation setting is replicated R=400 times. The average FP proportions are calculated for homoscedastic variance, where the heteroscedastic parameter vector $\gamma_{0}=0$. For heteroscedastic variance, where $\gamma_{0}$ has five nonnull components (3,1, −5, −5, −3), the average FP and TP proportions are calculated.

**Table 4.** Test (α=0.05) results are presented for the decorrelation procedure under both homoscedastic and heteroscedastic variance

	$\alpha =0.05,\tau_{1}=0.6,\tau_{2}=0.8$											
ε	ρ=0.3						ρ=0.5					
	Homoscedastic		Heteroscedastic				Homoscedastic		Heteroscedastic			
	N(0,1)	$t_{3}$	N(0,1)	$t_{3}$	N(0,1)		N(0,1)	$t_{3}$	N(0,1)	$t_{3}$	N(0,1)	$t_{3}$
	FP(size)		FP(size)		TP(power)		FP(size)		FP(size)		TP(power)	
High-sparsity	0.05	0.06	0.05	0.06	0.16	0.12	0.06	0.05	0.05	0.05	0.13	0.10
Medium-sparsity	0.06	0.05	0.06	0.06	0.08	0.09	0.07	0.07	0.06	0.07	0.07	0.08

*Note:* The pair of expectile levels is $\left(\tau_{1},\tau_{2}\right)=(0.6,0.8)$. The covariance matrix for $\tilde{X}\sim N(0,\tilde{𝚺})$ has components ${\tilde{𝚺}}_{ij}=\rho^{\left|i-j\right|}$ for i,j=1,…,p. The error distributions considered are N(0,1) and $t_{3}$. Each simulation setting is replicated R=400 times. The average FP proportions are calculated for homoscedastic variance, where the heteroscedastic parameter vector $\gamma_{0}=0$. For heteroscedastic variance, where $\gamma_{0}$ has five nonnull components (3,1, −5, −5, −3), the average FP and TP proportions are calculated.

## APPENDIX

### A. ASSUMPTIONS

For the sake of completeness, we summarize the assumptions used in our theory.

(A1) $\varepsilon_{1},\ldots ,\varepsilon_{n}$ are i.i.d. random variables with mean zero, a finite (2κ-2)th moment for κ≥2.
(A2) The empirical measure of the components of the p-vector $\gamma_{0}$, when p→∞, converges weakly to the probability measure of a random variable $\Gamma_{0}$ with bounded (2κ-2)th moment for κ≥2.
(A3) The empirical measure of the components of the p-vector $\beta_{0}$, when p→∞, converges weakly to the probability measure of a random variable $B_{0}$ with bounded (2κ-2)th moment for κ≥2.
(A4) A standard Gaussian design: $X_{i},i=1,\ldots ,n$, are i.i.d. copies of $X\in R^{p}$ with $X_{j}\sim N(0,1/n),j=1,\ldots ,p$, i.i.d. components.
(A5) For some κ>1,
  - ${\text{lim}}_{p\to \infty }E_{{\hat{f}}_{\beta_{0}(p)}}\left(B_{0}^{2\kappa -2}\right)=E_{f_{B_{0}}}\left(B_{0}^{2\kappa -2}\right)<\infty$;
  - ${\text{lim}}_{p\to \infty }E_{{\hat{f}}_{\gamma_{0}(p)}}\left(\Gamma_{0}^{2\kappa -2}\right)=E_{f_{\Gamma_{0}}}\left(\Gamma_{0}^{2\kappa -2}\right)<\infty$;
  - ${\text{lim}}_{p\to \infty }E_{{\hat{f}}_{\varepsilon (p)}}\left(\varepsilon^{2\kappa -2}\right)=E_{f_{\varepsilon }}\left(\varepsilon^{2\kappa -2}\right)<\infty$;
  - ${\text{lim}}_{p\to \infty }E_{{\hat{f}}_{q_{k}(p)}}\left(B_{k}^{2\kappa -2}\right)<\infty$.

### B. PROOFS

#### B.1. Proof of (3.10)

**Proof.** The proximal operator is defined as the minimizer of the objective function $b\rho (x)+\frac{1}{2}(x-z{)}^{2}$, which, by elementary calculus, is the root of the subgradient of the objective function. The proximal operator is then obtained by solving an equation of x as follows:

$$b\partial \rho_{\tau }\left(x;u_{\tau }\right)+x-z=0,$$

where $\partial \rho_{\tau }$ is the gradient of the differentiable expectile loss function $\rho_{\tau }$ in (3.12). Calculations follow similar steps and arguments for the two cases, that is, $x\leq u_{\tau }$ and $x>u_{\tau }$. We present the calculation steps for $x\leq u_{\tau }$ in detail. When $x\leq u_{\tau },\partial \rho_{\tau }\left(x;u_{\tau }\right)=2(1-\tau )\left(x-u_{\tau }\right)$. Then, by plugging in $\partial \rho_{\tau }$ and rearranging $2b(1-\tau )\left(x-u_{\tau }\right)+x-z=0$, we obtain

$$x=\frac{z+2b(1-\tau )u_{\tau }}{2b(1-\tau )+1}.$$

The domain of z is obtained by $\frac{z+2b(1-\tau )u_{\tau }}{2b(1-\tau )+1}\leq u_{\tau }$ which is $z\leq u_{\tau }$. □

#### B.2. Proof of (3.16)

**Proof.** By (3.14), the subgradient of the effective score function ${\tilde{G}}_{\tau }(z;b)$ in (3.13) w.r.t. z follows

$$\partial_{1}G_{\tau }\left(z;b\right)=\frac{\delta }{\omega }\left\{\frac{2b\left(1-\tau \right)}{2b\left(1-\tau \right)+1}I\left\{z\leq u_{\tau }\right\}+\frac{2b\tau }{2b\tau +1}I\left\{z>u_{\tau }\right\}\right\}.$$

The parameter b is updated by finding solutions b>0, s.t.

$$1=\frac{1}{n}\sum_{i=1}^{n}\partial_{1}G_{\tau }\left(z_{i};b\right)=\frac{\delta }{\omega }\left\{\frac{1}{n}\sum_{i=1}^{n}\frac{2b(1-\tau )}{2b(1-\tau )+1}I\left\{z_{i}\leq u_{\tau }\right\}+\frac{2b\tau }{2b\tau +1}I\left\{z_{i}>u_{\tau }\right\}\right\}.$$

□

#### B.3. Proof of Lemma 2

**Proof.** We first take ${\tilde{\psi }}_{c}$ in (3.26) to be ${\tilde{\psi }}_{c}\left(x_{1},\ldots ,x_{t+1},y\right)=\tilde{\psi }\left(x_{t+1},y\right)$. Then, (3.32) can be obtained by taking $\tilde{\psi }\left(x_{t+1},y\right)=\psi \left(y-x_{t+1}\right)$ and using (3.25). □

#### B.4. Proof of Theorem 1

We first prove (3.29) and (3.30). By choosing ${\tilde{\psi }}_{c}\left(x_{1},\ldots ,x_{t+1},y\right)=-x$ in (3.26), we obtain

$$\underset{p\to \infty }{\text{lim}}\frac{1}{p}\left({\tilde{\beta }}_{k_{1},(t+1),j}-\beta_{k_{1},j}\right)\left({\tilde{\beta }}_{k_{2},(t+1),j}-\beta_{k_{2},j}\right)\overset{\text{a.s.}}{=}E\left[\left(-{\overset{‾}{\zeta }}_{k_{1},(t)}Z_{k_{1},(t)}\right)\left(-{\overset{‾}{\zeta }}_{k_{2},(t)}Z_{k_{2},(t)}\right)\right]={\overset{‾}{\zeta }}_{k_{1},(t)}{\overset{‾}{\zeta }}_{k_{2},(t)}E\left[Z_{k_{1},(t)}Z_{k_{2},(t)}\right]={\overset{‾}{\zeta }}_{k_{1},(t)}{\overset{‾}{\zeta }}_{k_{2},(t)}\text{Cov}\left(Z_{k_{1}},Z_{k_{2}}\right).$$

The last equality holds since $\left(Z_{k_{1},(t)},Z_{k_{2},(t)}\right)$ is a standard normal random 2-vector. And we drop the iteration index t for convenience.

Similarly, we choose ${\tilde{\psi }}_{c^{\prime }}\left(x_{1},\ldots ,x_{t},y\right)$ and ${\tilde{\psi }}_{c^{\prime \prime }}\left(x_{1},\ldots ,x_{t},y\right)$ in (3.27) to be the rescaled effective score functions $G_{k_{1}}\left(y-x_{t};\cdot \right)$ and $G_{k_{2}}\left(y-x_{t};\cdot \right)$. By (3.24), we obtain

$$\underset{n\to \infty }{\text{lim}}\frac{1}{n}\sum_{i=1}^{n}G_{k_{1}}\left(z_{k_{1},i,(t)};b_{k_{1},(t)}\right)G_{k_{2}}\left(z_{k_{2},i,(t)};b_{k_{2},(t)}\right)\;\overset{\text{a.s.}}{=}E\left[G_{k_{1}}\left(\varepsilon -{\overset{‾}{\sigma }}_{k_{1}}{\overset{ˆ}{Z}}_{k_{1}};b_{k_{1},(t)}\right)G_{k_{2}}\left(\varepsilon -{\overset{‾}{\sigma }}_{k_{2}}{\overset{ˆ}{Z}}_{k_{2}};b_{k_{2},(t)}\right)\right]\;=E\left[G_{k_{1}}\left(\varepsilon +{\overset{‾}{\sigma }}_{k_{1}}{\overset{ˆ}{Z}}_{k_{1}};b_{k_{1},(t)}\right)G_{k_{2}}\left(\varepsilon +{\overset{‾}{\sigma }}_{k_{2}}{\overset{ˆ}{Z}}_{k_{2}};b_{k_{2},(t)}\right)\right].$$

The last equality holds by $Z\overset{d}{=}-Z$ for Z~N(0,1).

To obtain (3.31), we first combine (3.24), (3.25), and (3.28) which leads to

$$\frac{1}{n}\sum_{i=1}^{n}G_{k_{1}}\left(z_{k_{1},i,(t)};b_{k_{1},(t)}\right)G_{k_{2}}\left(z_{k_{2},i,(t)};b_{k_{2},(t)}\right)\overset{P}{\to }\underset{p\to \infty }{\text{lim}}\frac{1}{p}\left({\tilde{\beta }}_{k_{1},(t+1),j}-\beta_{k_{1},j}\right)\left({\tilde{\beta }}_{k_{2},(t+1),j}-\beta_{k_{2},j}\right).$$

Then, when n,p are sufficiently large, we use (3.31) to approximate the covariance of ${\tilde{\beta }}_{k_{1},(t+1)}$ and ${\tilde{\beta }}_{k_{2},(t+1)}$.

#### B.5. Proof of Theorem 2

**Proof.**

$$\vartheta \left(\gamma_{0,j}\right)=I\left\{T_{j}\in R_{j},\Gamma_{0}=\gamma_{0,j}\right\}\;=I\left\{\frac{\Delta {\tilde{\xi }}_{j}^{⊤}}{\sqrt{\Delta \Sigma \Delta^{⊤}}}>\Phi^{-1}(1-\alpha /2)\right\}+I\left\{\frac{\Delta {\tilde{\xi }}_{j}^{⊤}}{\sqrt{\Delta \Sigma \Delta^{⊤}}}<\Phi^{-1}(\alpha /2)\right\}\;=I\left\{\frac{\Delta {\left({\tilde{\xi }}_{j}-\left(u_{\tau_{1}},u_{\tau_{2}}\right)\gamma_{0,j}\right)}^{⊤}}{\sqrt{\Delta \Sigma \Delta^{⊤}}}>\Phi^{-1}(1-\alpha /2)-\gamma_{0,j}\frac{\Delta {\left(u_{\tau_{1}},u_{\tau_{2}}\right)}^{⊤}}{\sqrt{\Delta \Sigma \Delta^{⊤}}}\right\}\;+I\left\{\frac{\Delta {\left({\tilde{\xi }}_{j}-\left(u_{\tau_{1}},u_{\tau_{2}}\right)\gamma_{0,j}\right)}^{⊤}}{\sqrt{\Delta \Sigma \Delta^{⊤}}}<\Phi^{-1}(\alpha /2)-\gamma_{0,j}\frac{\Delta {\left(u_{\tau_{1}},u_{\tau_{2}}\right)}^{⊤}}{\sqrt{\Delta \Sigma \Delta^{⊤}}}\right\}.$$

By Lemma 1 and define $\tilde{\Xi }=\left(B_{0}+u_{\tau_{1}}\Gamma_{0}+{\overset{‾}{\zeta }}_{1}Z_{1},B_{0}+u_{\tau_{2}}\Gamma_{0}+{\overset{‾}{\zeta }}_{2}Z_{2}\right)$, where $\left({\overset{‾}{\zeta }}_{1}Z_{1},{\overset{‾}{\zeta }}_{2}Z_{2}\right)\sim N(0,\Sigma )$, we obtain

$$\underset{p\to \infty }{\text{lim}}\frac{1}{p}\sum_{j=1}^{p}\vartheta \left(\gamma_{0,j}\right)\;\overset{\text{a.s.}}{=}EI\left\{\frac{\Delta {\left(\tilde{\Xi }-\left(u_{\tau_{1}},u_{\tau_{2}}\right)\Gamma_{0}\right)}^{⊤}}{\sqrt{\Delta \Sigma \Delta^{⊤}}}>\Phi^{-1}(1-\alpha /2)-\Gamma_{0}\frac{\Delta {\left(u_{\tau_{1}},u_{\tau_{2}}\right)}^{⊤}}{\sqrt{\Delta \Sigma \Delta^{⊤}}}\right\}\;+EI\left\{\frac{\Delta {\left(\tilde{\Xi }-\left(u_{\tau_{1}},u_{\tau_{2}}\right)\Gamma_{0}\right)}^{⊤}}{\sqrt{\Delta \Sigma \Delta^{⊤}}}<\Phi^{-1}(\alpha /2)-\Gamma_{0}\frac{\Delta {\left(u_{\tau_{1}},u_{\tau_{2}}\right)}^{⊤}}{\sqrt{\Delta \Sigma \Delta^{⊤}}}\right\}\;=EI\left\{Z_{3}>\Phi^{-1}(1-\alpha /2)-\Gamma_{0}\frac{\Delta {\left(u_{\tau_{1}},u_{\tau_{2}}\right)}^{⊤}}{\sqrt{\Delta \Sigma \Delta^{⊤}}}\right\}+EI\left\{Z_{3}<\Phi^{-1}(\alpha /2)-\Gamma_{0}\frac{\Delta {\left(u_{\tau_{1}},u_{\tau_{2}}\right)}^{⊤}}{\sqrt{\Delta \Sigma \Delta^{⊤}}}\right\}\;=E_{\Gamma_{0}}\left[1-\Phi \left(\Phi^{-1}(1-\alpha /2)-\Gamma_{0}\frac{\Delta {\left(u_{\tau_{1}},u_{\tau_{2}}\right)}^{⊤}}{\sqrt{\Delta \Sigma \Delta^{⊤}}}\right)+\Phi \left(\Phi^{-1}(\alpha /2)-\Gamma_{0}\frac{\Delta {\left(u_{\tau_{1}},u_{\tau_{2}}\right)}^{⊤}}{\sqrt{\Delta \Sigma \Delta^{⊤}}}\right)\right].$$

□

### C. AUXILIARY DEFINITIONS AND LEMMAS

Definition 1 (Pseudo Lipschitz function). *A function* $\phi :R^{m}\to R$ *is pseudo-Lipschitz of order* κ≥1, *if there exists a constant* L>0, *such that for all* $x,y\in R^{m}$,

$$|\phi (x)-\phi (y)|\leq L\left(1+\|x{\|}^{\kappa -1}+\|y{\|}^{\kappa -1}\right)\left\|x-y\right\|.$$

It follows that if ϕ is a pseudo Lipschitz function of order κ, then there exists a constant $L^{\prime }$ such that for all $x\in R^{m}:|\phi (x)|\leq L^{\prime }\left(1+\|x{\|}^{\kappa }\right)$.

Lemma 3. *For any pseudo Lipschitz functions* $\phi_{c^{\prime }},\phi_{c^{\prime \prime }}:R^{m}\to R$ *of order* $\kappa_{c^{\prime }}$ *and* $\kappa_{c^{\prime \prime }}$, *their product function* $\phi_{c^{\prime }}\phi_{c^{\prime \prime }}$ *is a pseudo Lipschitz function of order* $\kappa_{c^{\prime }}+\kappa_{c^{\prime \prime }}$.

Lemma 4 (Bayati and Montanari (2011a, Lemma 2)). *For any deterministic unit vectors* $u\in R^{p}$ *and* $v\in R^{n}$, *and a random matrix* $\tilde{X}$ *distributed as* $X\in R^{n\times p}$, *the following conclusions hold:*

1. $v^{⊤}\tilde{X}u\overset{d}{=}Z/\sqrt{n}$, *where* Z~N(0,1).
2. ${\text{lim}}_{n\to \infty }\|\tilde{X}u{\|}^{2}=1$ *almost surely*.
3. *Consider a* d-*dimensional subspace* $S\in R^{n}$ *with orthonormal basis* $s_{1},\ldots ,s_{d}$ *and* ${\left\|s_{i}\right\|}^{2}=n,i=1,\ldots ,d$. *Denote the matrix* $D=\left(s_{1},\ldots ,s_{d}\right)$ *and the orthogonal projection onto* S *as* $P_{S}$. *Then, we have* $P_{S}\tilde{X}u\overset{d}{=}Dx$ *with* $x\in R^{d}$ *satisfying* ${\text{lim}}_{n\to \infty }\|x\|\overset{\text{a.s.}}{=}0$ *and* $x=o_{d}(1)$.

Lemma 5 (Jameson (2014, Thm. 1)). *If* $x_{i}\geq 0$ *where* i=1,…,n *and* p≥1, *then* $\sum_{i=1}^{n}x_{i}^{p}\leq {\left(\sum_{i=1}^{n}x_{i}\right)}^{p}\leq n^{p-1}\sum_{i=1}^{n}x_{i}^{p}$. *The reversed inequality holds for* p∈(0,1).

Lemma 6 (Bayati and Montanari (2011a, Lemma 4)). *Let* κ≥2 *and a sequence of vectors* $\{v(N){\}}_{N\geq 0}$ *whose empirical distribution converges weakly to probability measure* $f_{V}$ *on* R *with bounded* κ*th moment; additionally, assume that* ${\text{lim}}_{n\to \infty }E_{{\hat{f}}_{v}}\left(V^{\kappa }\right)=E_{f_{V}}\left(V^{\kappa }\right)$. *Then for any pseudo Lipschitz function* ψ:R→R *of order* κ,

$$\underset{N\to \infty }{\text{lim}}\frac{1}{N}\sum_{j=1}^{N}\psi \left(v_{j}\right)\overset{\text{a.s.}}{=}E[\psi (V)].$$

Lemma 7. *For any fixed unit vectors* $u\in R^{p},v\in R^{n}$ *and random matrices* $\tilde{X}$ *distributed as* $X\in R^{n\times p}$, *it holds that* $\tilde{X}u={\left(\sum_{j=1}^{p}{\tilde{X}}_{1,j}u_{j},\ldots ,\sum_{j=1}^{p}{\tilde{X}}_{n,j}u_{j}\right)}^{⊤}\overset{d}{=}\frac{1}{\sqrt{n}}{\left(Z_{1},\ldots ,Z_{n}\right)}^{⊤}\sim N\left(0,\frac{1}{n}I_{n}\right)$, *where* $Z_{i}’\text{s}$ *are i.i.d. samples of a standard normal* N(0,1) *distributed random variable Z. Similarly*, ${\tilde{X}}^{⊤}v={\left(\sum_{i=1}^{n}{\tilde{X}}_{i,1}u_{i},\ldots ,\sum_{i=1}^{n}{\tilde{X}}_{i,p}u_{i}\right)}^{⊤}\overset{d}{=}\frac{1}{\sqrt{n}}{\left(Z_{1}^{\prime },\ldots ,Z_{p}^{\prime }\right)}^{⊤}\sim N\left(0,\frac{1}{n}I_{p}\right)$, *where* $Z_{j}^{\prime }’\text{s}$ *are i.i.d. samples of a standard normal* N(0,1) *distributed random variable* $Z^{\prime }$.

Lemma 8 (Shi et al. (2012, Thm. 5)). *Assume any* $m_{1},m_{2}\in R^{n}$ *and any random Gaussian matrix* $\tilde{X}$ *distributed as* $\tilde{X}\in R^{n\times p}$. *For any* ϵ∈(0,1), *if the inner product $\left\langle m_{1},m_{2}\right\rangle >0$*, *then with probability at least* $1-6\text{exp}\left(-\frac{n}{2}\left(\frac{\epsilon^{2}}{2}-\frac{\epsilon^{3}}{3}\right)\right)$, *the following holds:*

$$\frac{1+\epsilon }{1-\epsilon }\frac{\left\langle m_{1},m_{2}\right\rangle }{\left\|m_{1}\right\|\left\|m_{2}\right\|}-\frac{2\epsilon }{1-\epsilon }\leq \frac{\left\langle {\tilde{X}}^{⊤}m_{1},{\tilde{X}}^{⊤}m_{2}\right\rangle }{\left\|{\tilde{X}}^{⊤}m_{1}\right\|\left\|{\tilde{X}}^{⊤}m_{2}\right\|}\leq 1-\frac{\sqrt{1-\epsilon^{2}}}{1+\epsilon }+\frac{\epsilon }{1+\epsilon }+\frac{1-\epsilon }{1+\epsilon }\frac{\left\langle m_{1},m_{2}\right\rangle }{\left\|m_{1}\right\|\left\|m_{2}\right\|}.$$
