# Supplementary material for: HIGH-DIMENSIONAL NEWEY–POWELL TEST VIA APPROXIMATE MESSAGE PASSING
Source: Econ Theory (N Y). Author manuscript; Available in PMC 2026 Jul 21. (PMC13384483; doi:10.1017/s0266466626100450)
Supplement: Supplementary [file NIHMS2194701-supplement-Supplementary.pdf]

Supplement to “HIGH-DIMENSIONAL NEWEY-POWELL TEST VIA APPROXIMATE MESSAGE PASSING”

Jing Zhou * and Hui Zou**

University of Manchester and University of Minnesota Twin Cities

Equations, tables, and figures in this supplement are labeled with the prefix “S”. Without this prefix, references correspond to items in the main manuscript.

1 Proofs

1.1 Proof of Lemma 1

Proof. Lemma 1 generalizes Bayati and Montanari (2011, Proof of Lemma 1) by showing correlation properties for two sequences k_1, k_2. A similar proof strategy in Bayati and Montanari (2011, Proof of Lemma 1) is used by induction on the iteration index t. For $k = k_1, k_2$, let $\mathcal{B}_{k,(t)}$ be the properties for $d_{k,(t)}, q_{k,(t)}$ in Bayati and Montanari (2011, Eq. (3.15), (3.17), (3.19), (3.21), (3.23), (3.26)), and $\mathcal{H}_{k,(t+1)}$ be the properties for $m_{k,(t)}, h_{k,(t)}$ in Bayati and Montanari (2011, Eq. (3.14), (3.16), (3.18), (3.20), (3.22), (3.24), (3.25)). We assume that $\mathcal{B}_{k,(t)}$ and $\mathcal{H}_{k,(t+1)}$ hold for the chains k_1, k_2; this is a direct implication of Lemma 1 in Bayati and Montanari (2011). The proof consists of four steps

1. Assume $\mathcal{B}_{k_1,(0)}$ and $\mathcal{B}_{k_2,(0)}$ hold, then (3.27) holds for $t = 0$.

2. Assume $\mathcal{H}_{k_1,(1)}$ and $\mathcal{H}_{k_2,(1)}$ hold, then (3.26) and (3.28) hold for $t = 0$.

3. Assume $\mathcal{B}_{k_1,(t')}, \mathcal{B}_{k_2,(t')}$ hold. If (3.27) holds for $t' < t$, and $\mathcal{H}_{k_1,(t'')}, \mathcal{H}_{k_2,(t'')}$, (3.26), (3.28) hold for $t'' \le t$, then (3.27) holds for t.

4. Assume $\mathcal{B}_{k_1,(t')}, \mathcal{B}_{k_2,(t')}$ hold. If (3.27) holds for $t' \le t$, and $\mathcal{H}_{k_1,(t'')}, \mathcal{H}_{k_2,(t'')}$, (3.26), (3.28) hold for $t'' \le t$, then (3.26) and (3.28) hold for $t + 1$.

*Department of Mathematics, University of Manchester. Email: jing.zhou@manchester.ac.uk

**School of Statistics, University of Minnesota (Twin Cities). Email: zouxx019@umn.edu

Further, for $k = k_1, k_2$, we define $\mathcal{D}_{k,(t'),(t'')}$ to be the σ-algebra generated by $d_{k,(0)}, \dots, d_{k,(t'-1)}$, $m_{k,(0)}, \dots, m_{k,(t'-1)}$, $h_{k,(0)}, \dots, h_{k,(t'')}$, $q_{k,(0)}, \dots, q_{k,(t'')}$, $\boldsymbol{\varepsilon}$, and $\beta_k = \beta_0 + u_k\gamma_0$. Let $M_{k,(t)} = (m_{k,(0)}, \dots, m_{k,(t-1)})$ and $Q_{k,(t)} = (q_{k,(0)}, \dots, q_{k,(t-1)})$. We denote $m^{\parallel}_{k,(t)}$ the projection of $m_{k,(t)}$ onto the column space of M_t, which can be expressed as $m^{\parallel}_{k,(t)} = \sum_{r=0}^{t-1} \delta'_{k,r} m_{k,(r)}$ and $\delta'_{k,r}$'s are some fixed coefficients. Then, we define the orthogonal projection $m^{\perp}_{k,(t)} = m_{k,(t)} - m^{\parallel}_{k,(t)}$. Similarly, let $q^{\parallel}_{k,(t)}$ and $q^{\perp}_{k,(t)}$ be the parallel and orthogonal projections onto $Q_{k,(t)}$.

1. Step 1. Assume $\mathcal{B}_{k_1,(0)}$ and $\mathcal{B}_{k_2,(0)}$ hold.
   By Bayati and Montanari (2011, Proof of Lemma 1, Step 1 (a), (b)), for $k = k_1, k_2$, let $\mathcal{D}_{k,(0),(0)}$ be generated by β_k, $q_{k,(0)}$, $\boldsymbol{\varepsilon}$, then

$$d_{k,(0)} \mid \mathcal{D}_{k,(0),(0)} \overset{d}{=} \boldsymbol{X} q^{\perp}_{k,(0)}, \tag{S.1}$$

where the components $[\boldsymbol{X} q_{k,(0)}]_i \overset{d}{=} Z_k \|q_{k,(0)}\|/\sqrt{n}$. The standard normal random 2-vector (Z_{k_1}, Z_{k_2}) has a non-zero correlation due to the common design matrix $\boldsymbol{X}$. We first show that the following random variable satisfies the condition of the strong law of large numbers for triangular arrays, which is also stated in Bayati and Montanari (2011, Theorem 3)

$$\tilde{\psi}_{c'}(d_{k_1,(0),i}, \varepsilon_i)\tilde{\psi}_{c''}(d_{k_2,(0),i}, \varepsilon_i) - \mathbb{E}_{\boldsymbol{X}}[\tilde{\psi}_{c'}(d_{k_1,(0),i}, \varepsilon)\tilde{\psi}_{c''}(d_{k_2,(0),i}, \varepsilon)].$$

For $\rho \in (0,1)$, and we denote $c_{k,i} = ([\boldsymbol{X} q_{k,(0)}]_i, \varepsilon_i)$ and $\tilde{c}_{k,i} = ([\tilde{\boldsymbol{X}} q_{k,(0)}]_i, \varepsilon_i)$, $k = k_1, k_2$,

$$\begin{aligned}
&\mathbb{E}\big|\tilde{\psi}_{c'}(d_{k_1,(0),i}, \varepsilon_i)\tilde{\psi}_{c''}(d_{k_2,(0),i}, \varepsilon_i) - \mathbb{E}_{\boldsymbol{X}}[\tilde{\psi}_{c'}(d_{k_1,(0),i}, \varepsilon)\tilde{\psi}_{c''}(d_{k_2,(0),i}, \varepsilon)]\big|^{2+\rho} \\
=\ & \mathbb{E}_{\tilde{\boldsymbol{X}}}\left|\tilde{\psi}_{c'}(\tilde{c}_{k_1,i})\tilde{\psi}_{c''}(\tilde{c}_{k_2,i}) - \mathbb{E}_{\boldsymbol{X}}[\tilde{\psi}_{c'}(c_{k_1,i})\tilde{\psi}_{c''}(c_{k_2,i})\right|^{2+\rho} \\
=\ & \mathbb{E}_{\tilde{\boldsymbol{X}}}\mathbb{E}_{\boldsymbol{X}}|\tilde{\psi}_{c'}(\tilde{c}_{k_1,i})\tilde{\psi}_{c''}(\tilde{c}_{k_2,i}) - \tilde{\psi}_{c'}(c_{k_1,i})\tilde{\psi}_{c''}(c_{k_2,i})|^{2+\rho} \\
=\ & \mathbb{E}|\tilde{\psi}_{c'}(\tilde{c}_{k_1,i})\tilde{\psi}_{c''}(\tilde{c}_{k_2,i}) - \tilde{\psi}_{c'}(\tilde{c}_{k_1,i})\tilde{\psi}_{c''}(c_{k_2,i}) \\
&\qquad + \tilde{\psi}_{c'}(\tilde{c}_{k_1,i})\tilde{\psi}_{c''}(c_{k_2,i}) - \tilde{\psi}_{c'}(c_{k_1,i})\tilde{\psi}_{c''}(c_{k_2,i})|^{2+\rho} \\
\leq\ & 2^{2+\rho}\{\mathbb{E}|\tilde{\psi}_{c'}(\tilde{c}_{k_1,i})\tilde{\psi}_{c''}(\tilde{c}_{k_2,i}) - \tilde{\psi}_{c'}(\tilde{c}_{k_1,i})\tilde{\psi}_{c''}(c_{k_2,i})|^{2+\rho} \\
& + \mathbb{E}|\tilde{\psi}_{c'}(\tilde{c}_{k_1,i})\tilde{\psi}_{c''}(c_{k_2,i}) - \tilde{\psi}_{c''}(c_{k_1,i})\tilde{\psi}_{c''}(c_{k_1,i})|^{2+\rho}\}.
\end{aligned}$$

The first two equalities use (S.1); $\tilde{\boldsymbol{X}}$ is an independent copy of $\boldsymbol{X}$. The inequality holds by Lemma 5. Next, we only focus on discussing the first term

since the discussion for the second term follows the same arguments.

$$\begin{aligned}
&\mathbb{E}|\tilde{\psi}_{c'}(\tilde{A}_{k_1,i})\tilde{\psi}_{c''}(\tilde{A}_{k_2,i}) - \tilde{\psi}_{c'}(\tilde{A}_{k_1,i})\tilde{\psi}_{c''}(A_{k_2,i})|^{2+\rho} \\
&\leq \mathbb{E}\Big\{|\tilde{\psi}_{c'}(\tilde{A}_{k_1,i})||\tilde{\psi}_{c''}(\tilde{A}_{k_2,i}) - \tilde{\psi}_{c''}(A_{k_2,i})|\Big\}^{2+\rho} \\
&\leq \mathbb{E}\Big\{L_1\big(1+\|\tilde{A}_{k_1,i}\|^{\kappa_c'}\big) \qquad\qquad (S.2)\\
&\quad L_2\big(\|[\tilde{\boldsymbol{X}}q_{k_2,(0)}]_i - [\boldsymbol{X}q_{k_2,(0)}]_i\|\big)\big(\|\tilde{A}_{k_2,i}\|^{\kappa_c''-1} + \|\tilde{A}_{k_1,i}\|^{\kappa_c''-1}+1\big)\Big\}^{2+\rho}.
\end{aligned}$$

The expectation is w.r.t. $\tilde{\boldsymbol{X}}$ and $\boldsymbol{X}$. The expectation $\mathbb{E}|Xq_{k,(0)}|_i|^k = (\frac{\|q_{k,(0)}\|}{\sqrt{n}})^k\mathbb{E}|Z|^k$ is bounded by some constant for $k \geq 1$. Then, by grouping $[\tilde{\boldsymbol{X}}q_{k,(0)}]_i$'s and ε_i's, (S.2) can further be simplified as a sum of a constant and $|\varepsilon_i|^{k_c'+k_c''-1}$ scaled by some constant. Then,

$$\frac{1}{n}\sum_{i=1}^{n}\mathbb{E}|\tilde{\psi}_{c'}(\tilde{A}_{k_1,i})\tilde{\psi}_{c''}(\tilde{A}_{k_2,i}) - \tilde{\psi}_{c'}(\tilde{A}_{k_1,i})\tilde{\psi}_{c''}(A_{k_2,i})|^{2+\rho} \leq c'n^{\rho/2}$$

holds if $\frac{1}{n}\sum_{i=1}^{n}|\varepsilon_i|^{(k_c'+k_c''-1)(2+\rho)} = \frac{1}{n}\sum_{i=1}^{n}|\varepsilon_i|^{(\kappa-1)(2+\rho)} \leq c'n^{\rho/2}$ holds, which has been shown in Bayati and Montanari (2011, proof of Lemma 1(b), Step 1). Then, by applying Bayati and Montanari (2011, Theorem 3), we obtain

$$\lim_{n\to\infty}\frac{1}{n}\sum_{i=1}^{n}\Big\{\tilde{\psi}_{c'}(d_{k_1,(0),i},\varepsilon_i)\tilde{\psi}_{c''}(d_{k_2,(0),i},\varepsilon_i) - \mathbb{E}_{\boldsymbol{X}}[\tilde{\psi}_{c'}(d_{k_1,(0),i},\varepsilon)\tilde{\psi}_{c''}(d_{k_2,(0),i},\varepsilon)]\Big\} \stackrel{a.s.}{=} 0.$$

By taking $\psi(v) = \mathbb{E}_{\hat{Z}_{k_1},\hat{Z}_{k_2}}[\tilde{\psi}_{c'}(\hat{Z}_{k_1}\|q_{k_1,(0)}\|/\sqrt{n}, v)\tilde{\psi}_{c''}(\hat{Z}_{k_2}\|q_{k_2,(0)}\|/\sqrt{n}, v)]$, where $v = \varepsilon$ in Lemma 6, and noticing $\psi(v)$ is a pseudo-Lipschitz function with order $\kappa_{c'} + \kappa_{c''} = \kappa$ by Lemma 3, we obtain

$$\lim_{n\to\infty}\frac{1}{n}\sum_{i=1}^{n}\tilde{\psi}_{c'}(d_{k_1,(0),i},\varepsilon_i)\tilde{\psi}_{c''}(d_{k_2,(0),i},\varepsilon_i) \stackrel{a.s.}{=} \mathbb{E}_{\varepsilon}\mathbb{E}_{\hat{Z}_{k_1},\hat{Z}_{k_2}}[\tilde{\psi}_{c'}(\bar{\sigma}_{k_1,(0)}\hat{Z}_{k_1},\varepsilon)\tilde{\psi}_{c''}(\bar{\sigma}_{k_2,(0)}\hat{Z}_{k_2},\varepsilon)].$$

2. Step 2. Assume $\mathcal{H}_{k_1,(1)}$ and $\mathcal{H}_{k_2,(1)}$ hold.

   We first show that (3.28) holds. By Bayati and Montanari (2011, Eq.(3.35)), it holds that for $k = k_1, k_2$

$$h_{k,(1)} \mid \mathcal{D}_{k,(1),(0)} \stackrel{d}{=} \tilde{\boldsymbol{X}}m_{k,(0)} + o_1(1)q_{k,(0)},$$

   where $\tilde{\boldsymbol{X}} \stackrel{d}{=} \boldsymbol{X}$ is independent of $\mathcal{D}_{k,(1),(0)}$ and $\mathbb{E}[\boldsymbol{X} \mid \mathcal{D}_{k,(1),(0)}]$. Further, the error term $o_1(1)q_{k,(0)}$ can be dropped in calculations, see for example Bayati

and Montanari (2011, proof of Lemma 1(b), (c)), Zhou et al. (2020, proof of Lemma 1). We first show that the two limits $\lim_{p\to\infty}\frac{1}{p}\sum_{j=1}^{p} h_{k_1,(1),j}h_{k_2,(1),j}$ and $\lim_{n\to\infty}\frac{1}{n}\sum_{i=1}^{n} m_{k_1,(0),i}m_{k_2,(0),i}$ are finite. Let $\theta_{m_{k_1,(0)}m_{k_2,(0)}}$ denote the angle between $m_{k_1,(0)}$ and $m_{k_2,(0)}$, then, with probability 1,

$$
\begin{aligned}
&\frac{1}{n}\sum_{i=1}^{n} m_{k_1,(0),i}m_{k_2,(0),i} = \frac{1}{n}\langle m_{k_1,(0)}, m_{k_2,(0)}\rangle \\
=\ & \frac{1}{n}\|m_{k_1,(0)}\|\|m_{k_2,(0)}\|\cos\theta_{m_{k_1,(0)}m_{k_2,(0)}} \le \frac{1}{n}\|m_{k_1,(0)}\|\|m_{k_2,(0)}\| \\
=\ & \frac{\|m_{k_1,(0)}\|}{\sqrt{n}}\frac{\|m_{k_2,(0)}\|}{\sqrt{n}} \to \bar{\zeta}_{k_1,(0)}\bar{\zeta}_{k_2,(0)}, n\to\infty,
\end{aligned}
\tag{S.3}
$$

where $\bar{\zeta}_{k_1,(0)}$, $\bar{\zeta}_{k_2,(0)}$ are the square-root of the state evolution parameters in (3.19), (3.20) and are finite.

Conditional on $\mathcal{D}_{k_1,(1),(0)} \vee \mathcal{D}_{k_2,(1),(0)}$,

$$
\begin{aligned}
&\frac{1}{p}\sum_{j=1}^{p} h_{k_1,(1),j}h_{k_2,(1),j} \\
\stackrel{d}{=}\ & \frac{1}{p}\sum_{j=1}^{p}[\tilde{\boldsymbol{X}}^\top m_{k_1,(0)}]_j[\tilde{\boldsymbol{X}}^\top m_{k_2,(0)}]_j = \frac{1}{p}\langle \tilde{\boldsymbol{X}}^\top m_{k_1,(0)}, \tilde{\boldsymbol{X}}^\top m_{k_2,(0)}\rangle.
\end{aligned}
\tag{S.4}
$$

We first argue that $\lim_{p\to\infty}\frac{1}{p}\sum_{j=1}^{p} h_{k_1,(1),j}h_{k_2,(1),j} < \infty$ almost surely.

$$
\begin{aligned}
&\frac{1}{p}\langle \tilde{\boldsymbol{X}}^\top m_{k_1,(0)}, \tilde{\boldsymbol{X}}^\top m_{k_2,(0)}\rangle \\
\le\ & \frac{1}{p}\|\tilde{\boldsymbol{X}}^\top m_{k_1,(0)}\|\|\tilde{\boldsymbol{X}}^\top m_{k_2,(0)}\| = \frac{\|m_{k_1,(0)}\|}{\sqrt{n}}\frac{\|m_{k_2,(0)}\|}{\sqrt{n}}, p\to\infty.
\end{aligned}
\tag{S.5}
$$

The last equality holds with probability 1 by Lemma 4 and by (S.3), the above quantity is finite almost surely and converges to $\bar{\zeta}_{k_1,(0)}\bar{\zeta}_{k_2,(0)}$. A similar argument is stated in Bayati and Montanari (2011, Step 2 (c)). Next, we prove that the difference between $\frac{1}{p}\sum_{j=1}^{p} h_{k_1,(1),j}h_{k_2,(1),j}$ and $\frac{1}{n}\sum_{i=1}^{n} m_{k_1,(0),i}m_{k_2,(0),i}$ is negligible when $n,p\to\infty$. By Lemma 8, with probability at least $1-6\exp(-\frac{n}{2}(\frac{\epsilon_h^2}{2}-\frac{\epsilon_h^3}{3}))$, the following holds

$$
\begin{aligned}
&\frac{1}{p}\Big(\frac{1+\epsilon_h}{1-\epsilon_h}\frac{\langle m_{k_1,(0)}, m_{k_2,(0)}\rangle}{\|m_{k_1,(0)}\|\|m_{k_2,(0)}\|} - \frac{2\epsilon_h}{1-\epsilon_h}\Big) \\
&\le \frac{1}{p}\frac{\langle \tilde{\boldsymbol{X}}^\top m_{k_1,(0)}, \tilde{\boldsymbol{X}}^\top m_{k_2,(0)}\rangle}{\|\tilde{\boldsymbol{X}}^\top m_{k_1,(0)}\|\|\tilde{\boldsymbol{X}}^\top m_{k_2,(0)}\|} \\
&\le \frac{1}{p}\Big(1-\frac{\sqrt{1-\epsilon_h^2}}{1+\epsilon_h}+\frac{\epsilon_h}{1+\epsilon_h}+\frac{1-\epsilon_h}{1+\epsilon_h}\frac{\langle m_{k_1,(0)}, m_{k_2,(0)}\rangle}{\|m_{k_1,(0)}\|\|m_{k_2,(0)}\|}\Big),
\end{aligned}
\tag{S.6}
$$

where $\epsilon_h > 0$. We first address the lower bound of (S.4), which directly follows the lower bound of (S.6) as follows

$$\begin{aligned}
&\lim_{p\to\infty} \frac{1}{p}\Big(\frac{1+\epsilon_h}{1-\epsilon_h}\frac{\langle m_{k_1,(0)}, m_{k_2,(0)}\rangle}{\|m_{k_1,(0)}\|\|m_{k_2,(0)}\|} - \frac{2\epsilon_h}{1-\epsilon_h}\Big)\|\tilde{\boldsymbol{X}}^\top m_{k_1,(0)}\|\|\tilde{\boldsymbol{X}}^\top m_{k_2,(0)}\| \\
= &\lim_{p\to\infty}\left\{\frac{1+\epsilon_h}{1-\epsilon_h}\frac{1}{n}\langle m_{k_1,(0)}, m_{k_2,(0)}\rangle \frac{\|\tilde{\boldsymbol{X}}^\top m_{k_1,(0)}\|/\sqrt{p}}{\|m_{k_1,(0)}\|/\sqrt{n}}\frac{\|\tilde{\boldsymbol{X}}^\top m_{k_2,(0)}\|/\sqrt{p}}{\|m_{k_2,(0)}\|/\sqrt{n}} \right.\\
&\left. -\frac{2\epsilon_h}{1-\epsilon_h}\frac{\|\tilde{\boldsymbol{X}}^\top m_{k_1,(0)}\|}{\sqrt{p}}\frac{\|\tilde{\boldsymbol{X}}^\top m_{k_2,(0)}\|}{\sqrt{p}}\right\} \\
\overset{a.s.}{=} &\frac{1+\epsilon_h}{1-\epsilon_h}\frac{1}{n}\langle m_{k_1,(0)}, m_{k_2,(0)}\rangle - \frac{2\epsilon_h}{1-\epsilon_h}\frac{\|m_{k_1,(0)}\|}{\sqrt{n}}\frac{\|m_{k_2,(0)}\|}{\sqrt{n}},
\end{aligned} \tag{S.7}$$

where the first almost sure convergence holds by Lemma 4. The upper bound of (S.4) follows similarly, and we obtain

$$\begin{aligned}
&\lim_{p\to\infty}\frac{1}{p}\sum_{j=1}^{p} h_{k_1,(1),j}h_{k_2,(1),j} \\
\le &\frac{1-\epsilon_h}{1+\epsilon_h}\frac{1}{n}\langle m_{k_1,(0)}, m_{k_2,(0)}\rangle + \Big(1-\frac{\sqrt{1-\epsilon_h^2}}{1+\epsilon_h}+\frac{\epsilon_h}{1+\epsilon_h}\Big)\frac{\|m_{k_1,(0)}\|}{\sqrt{n}}\frac{\|m_{k_2,(0)}\|}{\sqrt{n}}.
\end{aligned} \tag{S.8}$$

Combining (S.6), (S.7), (S.8), and let $\epsilon_h = O(n^{-1/2+\delta})$ for some $\delta \in (0, 1/2)$, we obtain

$$\frac{1}{n}m_{k_1,(0),i}m_{k_2,(0),i} \xrightarrow{P} \lim_{p\to\infty}\frac{1}{p}\sum_{j=1}^{p} h_{k_1,(1),j}h_{k_2,(1),j}, \tag{S.9}$$

where $\xrightarrow{P}$ denotes convergence in probability.

The proof for (3.26) is similar to that for (3.27) and has been shown in Zhou et al. (2020, Section B.2.5) for homoscedastic linear models where $\beta_{k_1} = \beta_{k_2} = \beta_0$. We only show the proof for (3.26) in Step 2 to avoid being repetitive. Similar to Step 1, we could show that the condition in Bayati and Montanari (2011, Theorem 3) holds, resulting in the following convergence

$$\begin{aligned}
\lim_{p\to\infty}\frac{1}{p}\sum_{j=1}^{p}\Big\{&\tilde{\psi}_{\mathrm{c}}(h_{k_1,(1),j}, \beta_{k_1,j})\tilde{\psi}_{\mathrm{c}}(h_{k_2,(1),j}, \beta_{k_2,j}) \\
&-\mathbb{E}_{\tilde{X}}[\tilde{\psi}_{\mathrm{c}}(h_{k_1,(1),j}, \beta_{k_1,j})\tilde{\psi}_{\mathrm{c}}(h_{k_2,(1),j}, \beta_{k_2,j})]\Big\} \overset{\text{a.s.}}{=} 0.
\end{aligned}$$

Then, using Lemma 6 for $v(u) = \beta_0 + u\gamma_0$ and

$$\psi(v) = \mathbb{E}_{\tilde{X}}\tilde{\psi}_{\mathrm{c}}(h_{k_1,(1),j}, v(u_{k_1}))\tilde{\psi}_{\mathrm{c}}(h_{k_2,(1),j}, v(u_{k_2})),$$

the following convergence holds

$$\lim_{p\to\infty}\frac{1}{p}\sum_{j=1}^{p}\mathbb{E}_{\tilde{X}}\Big[\tilde{\psi}_{\mathrm{c}}(h_{k_1,(1),j},\beta_{0,j}+u_{k_1}\gamma_{0,j})\tilde{\psi}_{\mathrm{c}}(h_{k_2,(1),j},\beta_{0,j}+u_{k_2}\gamma_{0,j})\Big]$$
$$\stackrel{\text{a.s.}}{=}\ \mathbb{E}_{B_0}\Big[\mathbb{E}_{(Z_{k_1,(0)},Z_{k_2,(0)})}\Big[\tilde{\psi}_{\mathrm{c}}(\|\frac{m_{k_r,(0)}}{\sqrt{n}}\|Z_{k_1,(0)},B_0+u_{k_1}\Gamma_0)$$
$$\tilde{\psi}_{\mathrm{c}}(\|\frac{m_{k_r,(0)}}{\sqrt{n}}\|Z_{k_2,(0)},B_0+u_{k_2}\Gamma_0)\Big]\Big].$$

3. Step 3. Assume $\mathcal{B}_{k_1,(t')},\mathcal{B}_{k_2,(t')}$ hold. If (3.27) holds for $t'<t$, and $\mathcal{H}_{k_1,(t'')},\mathcal{H}_{k_2,(t'')}$, (3.26), (3.28) hold for $t''\le t$.

   By Bayati and Montanari (2011, Lemma 1(b) in Step 4) with dropped error term, given $\mathcal{D}_{k_1,(t),(t)}\vee\mathcal{D}_{k_2,(t),(t)}=\sigma(\mathcal{D}_{k_1,(t),(t)}\bigcup\mathcal{D}_{k_2,(t),(t)})$, we have

$$\tilde{\psi}_{c'}(d_{k_1,(0),i},\ldots,d_{k_1,(t),i},\varepsilon_i)\tilde{\psi}_{c''}(d_{k_2,(0),i},\ldots,d_{k_2,(t),i},\varepsilon_i)\stackrel{d}{=}$$
$$\tilde{\psi}_{c'}(d_{k_1,(0),i},\ldots,d_{k_1,(t-1),i},\big[\textstyle\sum_{r=0}^{t-1}\delta_{k_1,r}d_{k_1,(r)}+\tilde{\boldsymbol{X}}q^{\perp}_{k_1,(t)}\big]_i,\varepsilon_i)$$
$$\tilde{\psi}_{c''}(d_{k_2,(0),i},\ldots,d_{k_2,(t-1),i},\big[\textstyle\sum_{r=0}^{t-1}\delta_{k_2,r}d_{k_2,(r),i}+\tilde{\boldsymbol{X}}q^{\perp}_{k_2,(t)}\big]_i,\varepsilon_i),$$

   where $\delta_{k,r}$'s are coefficients for $d_{k,(r)}, r=1,\ldots,t-1$ for $k=k_1,k_2$. We define

$$c_{k,i}=(d_{k,(0),i},\ldots,d_{k,(t-1),i},\big[\sum_{r=0}^{t-1}\delta_{k,r}d_{k,(r)}+\tilde{\boldsymbol{X}}q^{\perp}_{k,(t)}\big]_i,\varepsilon_i).$$

   By Bayati and Montanari (2011, Theorem 3), we obtain

$$\lim_{n\to\infty}\frac{1}{n}\sum_{i=1}^{n}\Big[\tilde{\psi}_{c'}(c_{k_1,i})\tilde{\psi}_{c''}(c_{k_2,i})-\mathbb{E}_{\tilde{\boldsymbol{X}}}\big[\tilde{\psi}_{c'}(c_{k_1,i})\tilde{\psi}_{c''}(c_{k_2,i})\big]\Big]\stackrel{a.s.}{=}0,\qquad\text{(S.10)}$$

   where $[\tilde{\boldsymbol{X}}q^{\perp}_{k,(t)}]_i\stackrel{d}{=}\hat{Z}_k\|q^{\perp}_{k,(t)}\|/\sqrt{n}$. Thus, (S.10) can further be expressed as

$$\lim_{n\to\infty}\frac{1}{n}\sum_{i=1}^{n}\Big[\tilde{\psi}_{c'}(c_{k_1,i})\tilde{\psi}_{c''}(c_{k_2,i})-\mathbb{E}_{(\hat{Z}_{k_1},\hat{Z}_{k_2})}\big[\tilde{\psi}_{c'}(\tilde{c}_{k_1,i})\tilde{\psi}_{c''}(\tilde{c}_{k_2,i})\big]\Big]\stackrel{a.s.}{=}0,$$

   where $\tilde{c}_{k,i}=(d_{k,(0),i},\ldots,d_{k,(t-1),i},[\sum_{r=0}^{t-1}\delta_{k,r}d_{k,(r)}+\hat{Z}_k\|q^{\perp}_{k,(t)}\|/\sqrt{n}]_i), k=k_1,k_2$. And by Bayati and Montanari (2011, proof of Lemma 1(b) for Step 3), we know that $d_{k,(t),i}$'s are Gaussian distributed random variables with variance $\bar{\sigma}_{k,(t)}$. Then, the following convergence holds

$$\lim_{n\to\infty}\frac{1}{n}\sum_{i=1}^{n}\Big[\tilde{\psi}_{c'}(c_{k_1,i})\tilde{\psi}_{c''}(c_{k_2,i})$$
$$-\mathbb{E}_{\hat{Z}}\big[\tilde{\psi}_{c'}(\bar{\sigma}_{k_1,(0)}\hat{Z}_{k_1,(0)},\bar{\sigma}_{k_1,(t)}\hat{Z}_{k_1,(t)},\varepsilon)\tilde{\psi}_{c''}(\bar{\sigma}_{k_2,(0)}\hat{Z}_{k_2,(0)},\bar{\sigma}_{k_2,(t)}\hat{Z}_{k_2,(t)},\varepsilon)\big]\Big]\stackrel{a.s.}{=}0.$$

Lastly, by applying Lemma 6, we complete the proof showing (3.27) holds for iteration t.

4. Step 4. Assume $\mathcal{B}_{k_1,(t')}, \mathcal{B}_{k_2,(t')}$ hold. If (3.27) holds for $t' \leq t$, and $\mathcal{H}_{k_1,(t'')}, \mathcal{H}_{k_2,(t'')}$, (3.26), (3.28) hold for $t'' \leq t$.
Since the proof for (3.26) in Step 4 is similar to that for (3.27), and the complete proof for the homoscedastic regression models has been presented in Zhou et al. (2020, Proof of Lemma 1), the readers are suggested to refer to Step 2 for the main compositions of the proof. Next, we focus on presenting the detailed proof for (3.28). Given $\mathcal{D}_{k_1,(t+1),(t)} \vee \mathcal{D}_{k_2,(t+1),(t)}$, and by Bayati and Montanari (2011, Lemma 1(a), (b) in Step 4), we have

$$h_{k,(t+1)} \stackrel{d}{=} \sum_{r=0}^{t} \delta'_{k,(r)} h_{k,(r)} + \tilde{\boldsymbol{X}}^{\top} m^{\perp}_{k,(t)}, k = k_1, k_2. \tag{S.11}$$

By induction, for $t_1, t_2 < t$, the following holds

$$\frac{1}{n}\sum_{i=1}^{n} m_{k_1,(t_1),i} m_{k_2,(t_2),i} \xrightarrow{P} \lim_{p\to\infty} \frac{1}{p}\sum_{j=1}^{p} h_{k_1,(t_1+1),j} h_{k_2,(t_2+1),j}.$$

Further, the correlation between $m_{k_1,(t)}$ and $m_{k_2,(t)}$ is mainly caused by the common components β_0, γ_0, $\boldsymbol{\varepsilon}$, and $\boldsymbol{X}$ which enters $m^{\|}_{k,(t)} = \sum_{r=0}^{t-1} \delta'_{k,r} m_{k,(r)}$ at $t = 0$. In addition, at each iteration step $t = 1, \ldots$, a common independent Gaussian random matrix $\tilde{\boldsymbol{X}}$ brings in correlation between $m^{\perp}_{k_1,(t)} = m_{k_1,(t)} - m^{\|}_{k_1,(t)}$ and $m^{\perp}_{k_2,(t)} = m_{k_2,(t)} - m^{\|}_{k_2,(t)}$. Thus, we argue the validity of the following independence, i.e., $m^{\perp}_{k_1,(t)} \perp m_{k_2,(t'')}$ and $m^{\perp}_{k_2,(t)} \perp m_{k_1,(t'')}$ for $t'' < t$, since $m_{k,(t'')}$ is a vector forming $m^{\|}_{k,(t)}$.

Then, we show that (3.28) holds for $t_1 = t$ and $t_2 < t$. By induction hypothesis and the

$$
\begin{aligned}
&\frac{1}{n}\sum_{i=1}^{n} m_{k_1,(t),i} m_{k_2,(t_2),i} \\
&= \frac{1}{n}\sum_{i=1}^{n} m^{\|}_{k_1,(t),i} m_{k_2,(t_2),i} = \sum_{r=0}^{t} \delta'_{k_1,(r)} \frac{1}{n}\sum_{i=1}^{n} m_{k_1,(r),i} m_{k_2,(t_2),i} \\
&\xrightarrow{P} \sum_{r=0}^{t} \delta'_{k_1,(r)} \lim_{p\to\infty} \frac{1}{p}\sum_{j=1}^{p} h_{k_1,(r+1),j} h_{k_2,(t_2+1),j} \\
&= \lim_{p\to\infty} \frac{1}{p}\sum_{j=1}^{p} \big[\sum_{r=0}^{t} \delta'_{k_1,(r)} h_{k_1,(r+1)}\big]_j \big[h_{k_2,(t_2+1)}\big]_j \\
&\quad + \lim_{p\to\infty} \frac{1}{p}\sum_{j=1}^{p} [\tilde{\boldsymbol{X}}^\top m^{\perp}_{k_1,(t)}]_j \big[h_{k_2,(t_2+1)}\big]_j \\
&\stackrel{d}{=} \lim_{p\to\infty} \frac{1}{p}\sum_{j=1}^{p} \big[\sum_{r=0}^{t} \delta'_{k_1,(r)} h_{k_1,(r+1)} + \tilde{\boldsymbol{X}}^\top m^{\perp}_{k_1,(t)}\big]_j \big[h_{k_2,(t_2+1)}\big]_j \\
&= \lim_{p\to\infty} \frac{1}{p}\sum_{j=1}^{p} h_{k_1,(t+1),j} h_{k_2,(t_2+1),j}.
\end{aligned}
$$

The first equality holds by the independence of $m^{\perp}_{k_1,(t)}$ and $m_{k_2,(t_2)}$ for $t_2 < t$; the second equality holds by the definition of $m^{\|}_{k,(t)}$; the convergence in probability holds by induction hypothesis; the fourth equality holds since the second term is null due to the independence of $\tilde{\boldsymbol{X}}^\top m^{\perp}_{k_1,(t)}$ and $h_{k_2,(t_2+1)}$ (see the argument below (S.11) for detail); the equality in distribution is obtained by (S.11).

To complete the proof, we now show that (3.28) holds for $t_1, t_2 = t$. The proof technique combines (S.9) and that for $t_1 = t, t_2 < t$.

$$
\begin{aligned}
&\lim_{p\to\infty} \frac{1}{p}\sum_{j=1}^{p} h_{k_1,(t+1),j} h_{k_2,(t+1),j} \qquad \text{(S.12)} \\
&\stackrel{d}{=} \lim_{p\to\infty} \frac{1}{p}\sum_{j=1}^{p} \big[\sum_{r=0}^{t} \delta'_{k_1,(r)} h_{k_1,(r+1)} + \tilde{\boldsymbol{X}}^\top m^{\perp}_{k_1,(t)}\big]_j \big[\sum_{r=0}^{t} \delta'_{k_2,(r)} h_{k_2,(r+1)} + \tilde{\boldsymbol{X}}^\top m^{\perp}_{k_2,(t)}\big]_j \\
&= \lim_{p\to\infty} \Big\{ \sum_{r,r'=0}^{t} \delta'_{k_1,(r)} \delta'_{k_2,(r')} \frac{1}{p}\sum_{j=1}^{p} h_{k_1,(r+1),j} h_{k_2,(r'+1),j} \\
&\qquad + \frac{1}{p}\sum_{j=1}^{p} [\tilde{\boldsymbol{X}}^\top m^{\perp}_{k_1,(t)}]_j [\tilde{\boldsymbol{X}}^\top m^{\perp}_{k_2,(t)}]_j \Big\}.
\end{aligned}
$$

The first equivalence in distribution is by (S.11). The second and third terms on the right-hand side of the second equality vanished in the third equality due

to the independence of $\tilde{\boldsymbol{X}}^\top m^\perp_{k,(t)}$ and $h_{k',(r+1)}$, $k, k' = k_1, k_2$, $r = 0, \ldots, t$ (see the argument below (S.11) for detail). We now address the sequence

$$\begin{aligned} &\frac{1}{n}\sum_{i=1}^n m_{k_1,(t),i} m_{k_2,(t),i} \qquad\qquad (S.13) \\ &= \frac{1}{n}\sum_{i=1}^n m^{\|}_{k_1,(t),i} m^{\|}_{k_2,(t),i} + \frac{1}{n}\sum_{i=1}^n m^{\perp}_{k_1,(t),i} m^{\perp}_{k_2,(t),i} \\ &\overset{a.s.}{=} \sum_{r,r'=0}^t \delta'_{k_1,(r)}\delta'_{k_2,(r')}\frac{1}{n}\sum_{i=1}^n m_{k_1,(r),i} m_{k_2,(r'),i} \\ &\qquad + \lim_{p\to\infty}\frac{1}{p}\sum_{j=1}^p [\tilde{\boldsymbol{X}}^\top m^\perp_{k_1,(t)}]_j [\tilde{\boldsymbol{X}}^\top m^\perp_{k_2,(t)}]_j. \end{aligned}$$

The first equality holds by the definition of $m_{k,(t)}$. The first term $\frac{1}{n}\sum_{i=1}^n m^{\|}_{k_1,(t),i} m^{\|}_{k_2,(t),i}$ in the right-hand-side of the first equality, by the definition of $m^{\|}_{k,(t)}$, can be written as

$$\sum_{r,r'=0}^t \delta'_{k_1,(r)}\delta'_{k_2,(r')}\frac{1}{n}\sum_{i=1}^n m_{k_1,(r),i} m_{k_2,(r'),i}.$$

The almost sure convergence, by Lemma 4, holds for

$$\frac{1}{p}\sum_{j=1}^p [\tilde{\boldsymbol{X}}^\top m^\perp_{k_1,(t)}]_j [\tilde{\boldsymbol{X}}^\top m^\perp_{k_2,(t)}]_j.$$

By the induction hypothesis, the convergence in probability holds for the first term on the right-hand-side of the second equality of (S.13) and (S.12). And we conclude that

$$\frac{1}{n}\sum_{i=1}^n m_{k_1,(t),i} m_{k_2,(t),i} \xrightarrow{P} \lim_{p\to\infty}\frac{1}{p}\sum_{j=1}^p h_{k_1,(t+1),j} h_{k_2,(t+1),j}.$$

□

1.2 Proof of Lemma 3

Proof. For any pairs $x, y \in \mathbb{R}^m$,

$$\begin{aligned}
&|\phi_{c'}(x)\phi_{c''}(x) - \phi_{c'}(y)\phi_{c''}(y)| \\
= \;& |\phi_{c'}(x)\phi_{c''}(x) - \phi_{c'}(x)\phi_{c''}(y) + \phi_{c'}(x)\phi_{c''}(y) - \phi_{c'}(y)\phi_{c''}(y)| \\
\leq \;& |\phi_{c'}(x)\phi_{c''}(x) - \phi_{c'}(x)\phi_{c''}(y)| + |\phi_{c'}(x)\phi_{c''}(y) - \phi_{c'}(y)\phi_{c''}(y)| \\
\leq \;& |\phi_{c'}(x)||\phi_{c''}(x) - \phi_{c''}(y)| + |\phi_{c''}(y)||\phi_{c'}(x) - \phi_{c'}(y)| \\
\leq \;& 2\max\Big(L_1\|x-y\|(1+\|x\|^{\kappa_{c'}})(1+\|x\|^{\kappa_{c''}-1}+\|y\|^{\kappa_{c''}-1}), \\
& L_2\|x-y\|(1+\|x\|^{\kappa_{c''}})(1+\|x\|^{\kappa_{c'}-1}+\|y\|^{\kappa_{c'}-1})\Big) \\
\leq \;& \max\Big(L_1'\|x-y\|(1+\|x\|^{\kappa_{c''}}+\|y\|^{\kappa_{c''}})(1+\|x\|^{\kappa_{c'}-1}+\|y\|^{\kappa_{c'}-1}), \\
& L_2'\|x-y\|(1+\|x\|^{\kappa_{c''}-1}+\|y\|^{\kappa_{c''}-1})(1+\|x\|^{\kappa_{c'}}+\|y\|^{\kappa_{c'}})\Big) \\
\leq \;& \max\Big(L_1'\|x-y\|(1+\|x\|+\|y\|)^{\kappa_{c''}}(1+\|x\|+\|y\|)^{\kappa_{c'}-1}, \\
& L_2'\|x-y\|(1+\|x\|+\|y\|)^{\kappa_{c''}-1}\Big) \\
\leq \;& L''\|x-y\|(1+\|x\|+\|y\|)^{\kappa_{c'}+\kappa_{c''}-1} \\
\leq \;& L''3^{\kappa_{c'}+\kappa_{c''}-1}\|x-y\|(1+\|x\|^{\kappa_{c'}+\kappa_{c''}-1}+\|y\|^{\kappa_{c'}+\kappa_{c''}-1}).
\end{aligned}$$

The 5th and the last inequalities hold by Lemma 5. □

2 Numerical performance of the test using the estimator in (2.8)

Since estimation is more challenging when the error follows mixed normal distributions, we test the performance of the test with u_τ estimated by (2.8) in such settings. The estimator in (2.8) is only valid under the null. The performance of the test is reported in Table S.1.

$\alpha = 0.05$	$\tau_1 = 0.2, \tau_2 = 0.8$			$\tau_1 = 0.6, \tau_2 = 0.8$		
ε	$N(0,1)$	t_3	mixNormal 1	$N(0,1)$	t_3	mixnormal 1
High-sparsity	0.05	0.05	0.06	0.05	0.05	0.05
Medium-sparsity	0.05	0.05	0.06	0.05	0.05	0.06

Table S.1: Test results for ε following $N(0,1)$, t_3, $0.9N(-0.2, 0.25) + 0.1N(1.8, 0.01)$ (mixNormal 1) under homoscedastic variance. The nominal significance level $\alpha = 0.05$. The pairs of expectile levels are $(\tau_1, \tau_2) = (0.2, 0.8)$ on the left and $(\tau_1, \tau_2) = (0.6, 0.8)$ on the right. Each simulation setting is replicated for $R = 400$ times, and the averaged FP proportions are calculated.

3 Figure S.1

References

Bayati, M. and Montanari, A. (2011). The Dynamics of Message Passing on Dense Graphs, with Applications to Compressed Sensing. *IEEE Transactions on Information Theory*, 57(2):764–785.

Zhou, J., Claeskens, G., and Bradic, J. (2020). Detangling robustness in high dimensions: composite versus model-averaged estimation. *Electronic Journal of Statistics*, 14(2):2551–2599.

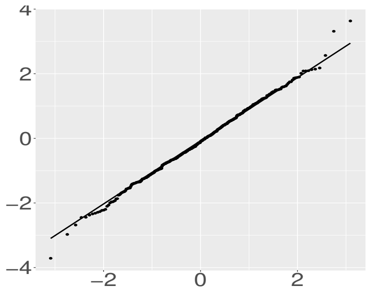

(a) High-sparsity $s = 5$; $\tau_1 = 0.1$.

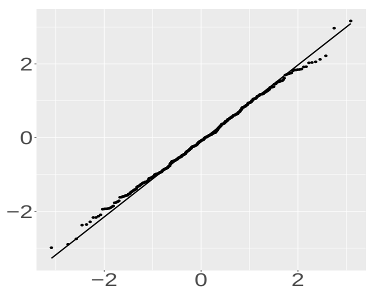

(b) High-sparsity $s = 5$; $\tau_1 = 0.2$.

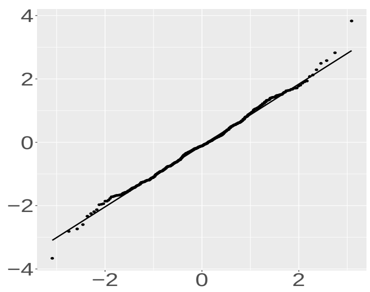

(c) High-sparsity $s = 5$; $\tau_1 = 0.6$.

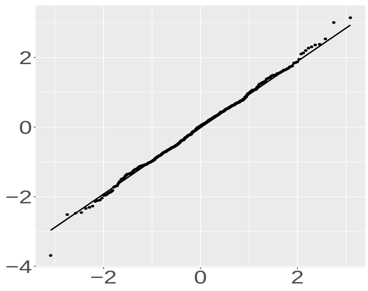

(d) Medium-sparsity $s = 40$; $\tau_1 = 0.1$.

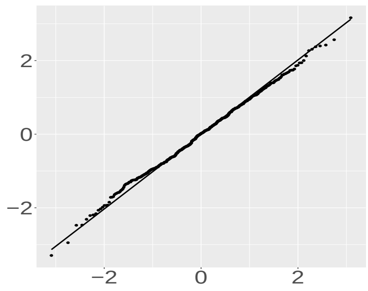

(e) Medium-sparsity $s = 40$; $\tau_1 = 0.2$.

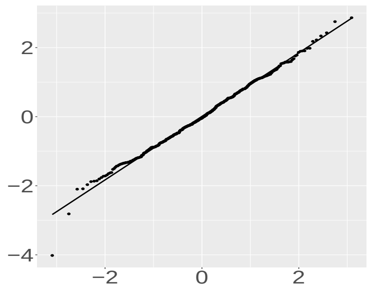

(f) Medium-sparsity $s = 40$; $\tau_1 = 0.6$.

Figure S.1: Normal QQ-plot of test statistics T_j's based on $\widetilde{\beta}$ at different expectile levels for three simulation settings. The test statistics T_j's should approximately follow a standard normal distribution $N(0,1)$ under homoscedasticity of $\boldsymbol{\varepsilon}$. The expectile levels fix $\tau_2 = 0.8$ and vary $\tau_1 = 0.1, 0.2, 0.6$; The left, middle, and right columns are plots for $\tau_1 = 0.1, 0.2, 0.6$, respectively. The high-sparsity and medium-sparsity plots are on the top and bottom rows, respectively.
